# Difficulty and prospects of endovascular treatment for spontaneous intracranial artery dissection

**DOI:** 10.3389/fneur.2025.1560883

**Published:** 2025-03-03

**Authors:** Lei Shi, Jinlu Yu

**Affiliations:** ^1^Department of Neurosurgery, The First Hospital of Jilin University, Changchun, China; ^2^Department of Neurosurgery, Henan Sanbo Brain Hospital, Zhengzhou, China

**Keywords:** intracranial artery dissection, endovascular treatment, prognosis, complication, review

## Abstract

Intracranial artery dissections (IADs) are relatively uncommon. For ruptured IADs and unruptured IADs with acute large artery occlusion, the size increases significantly during follow-up, or there are signs of compression with mass occupation. Intervention can be suggested. Currently, endovascular treatment (EVT) is the choice for treating IADs. However, the understanding of EVT for IADs remains limited; therefore, a thorough review is necessary on the basis of a literature review and our experience. In this review, the following issues are discussed: the incidence and natural history of IADs, angiography of IADs, EVT indications for IADs, EVT techniques to treat IADs, the prognosis and complications of EVT for IADs, and EVT techniques for each IAD. After reviewing the literature and on the basis of our experience, the review revealed that when IADs need intervention, deconstructive or reconstructive EVTs can be chosen as an effective option on case-by-case basis to achieve a good prognosis.

## 1 Introduction

Intracranial arteries are characterized by an absence of elastic fibers in the media, little adventitial tissue, no external elastic lamina and weaker supporting tissues (1). Therefore, after the internal elastic lamina is injured, the blood can invade the arterial wall through an entry to cause an intramural hematoma in the subintima, media or subadventitia, resulting in intracranial artery dissections (IADs) (2). IADs can occur at the location of intracranial arteries spontaneously or via trauma.

Spontaneous IADs may be underdiagnosed causes of stroke, which occurs in young and middle-aged East Asians (3). Most spontaneous IADs are asymptomatic. Symptomatic lesions can present with brain ischemia in 30–78% of cases, subarachnoid hemorrhage (SAH) in 50–60% of cases, and prodromal headache in 80% of cases (4). Uncommonly, large or giant lesions can cause brainstem or cranial nerve compression (1). Currently, the optimal management for spontaneous IADs is still unclear. Only certain IADs may need interventions, such as ruptured or symptomatic or progressive lesions.

IADs are difficult to treat with open surgery because of their broad and often shallow anatomical characteristics. Currently, endovascular treatment (EVT) plays an important role in treating IADs by restoring the lumen of the stenoses or occluded vessel, repairing a dilated thin artery or occluding the rupture point. However, EVT for IADs is challenging and complex due to its weak structure and distal location, among other factors. The current understanding of the utility of the EVT technique for spontaneous IADs is limited; therefore, a thorough review based on a literature review and our experience is necessary.

## 2 Methods and results of data collection

### 2.1 Literature search and strategy

In the review, the literature search was conducted in accordance with the Preferred Reporting Items for Systematic Reviews and Meta-Analyses 2020 statement (5). The PubMed database was searched for eligible studies written in English and published until February 1, 2025. On the PubMed website, the following keywords were input: (intracranial) AND [dissection(Title)] and (intracranial) AND [dissecting(Title)]. The types of eligible articles included case reports, case series, cohort studies, randomized controlled trials and prior systematic reviews and meta-analyses. The reference lists of the identified articles were also manually searched for additional significant articles that may have been missed. In addition, data from the past 5 years (February 1, 2020 to February 1, 2025) was procured in the database of our institute.

### 2.2 Inclusion and exclusion criteria

The inclusion criteria were as follows: articles about “spontaneous intracranial dissection” and articles for which the full text and sufficient information could be obtained. The exclusion criteria were as follows: articles without sufficient information and articles about traumatic intracranial dissection. In our database, inclusive cases need to have sufficient clinical, therapeutic and follow-up angiographic data.

### 2.3 Results

**Figure 1:**
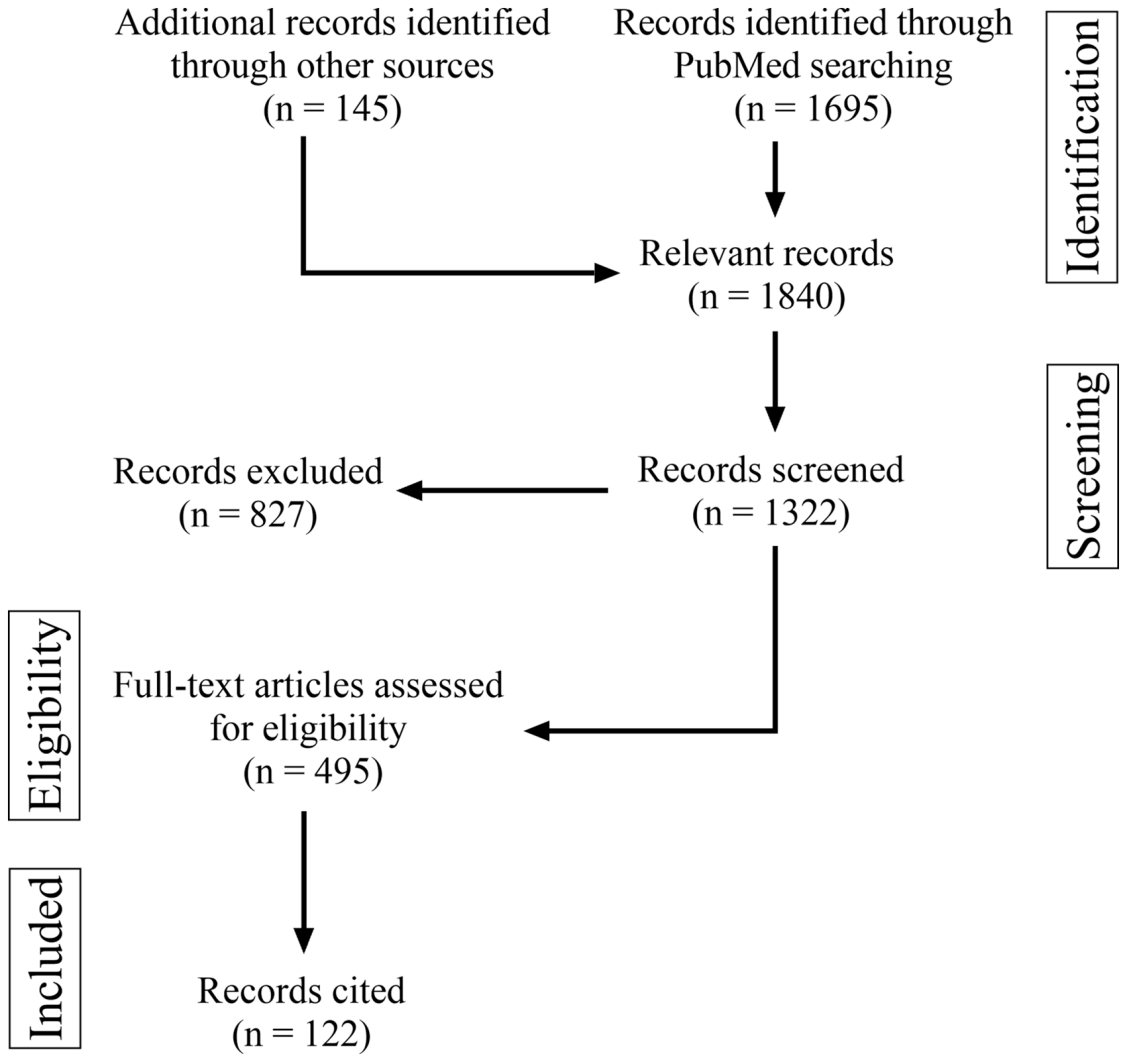
Flow chart of the literature search.

After screening and selecting eligible studies, 113 articles were included and cited. A flow chart displaying the literature collection process is shown in Figure 1. At our institute, 5,889 patients with intracranial vascular diseases who received EVT were identified and screened, and 650 spontaneous IADs were identified, and their data were read.

## 3 Incidence and natural history of IADs

### 3.1 Incidence

The incidence of spontaneous IADs is unknown (1, 6). Its proportion among all cranio-cervical dissections is estimated to be approximately 11% in European populations, 27% in Latin America, and 67–78% in East Asia (7–9). Most reports of spontaneous IADs are from Asia, and a male preponderance was noted (1). Spontaneous IADs tend to affect the posterior circulation more than the anterior circulation. The intracranial vertebral artery (VA) is the most common location (10). Among those that occur in the anterior circulation, the most common site is the middle cerebral artery (MCA) (11).

### 3.2 Natural history

The natural history of spontaneous IADs should vary. There should be differences between the anterior circulation and posterior circulation, main trunks and branches, proximal and distal segment of branches, large and small types, ruptured and unruptured types, fusiform and sidewall shapes, or flow-related or not. Until now, no reports with clear conclusions have been published. It was feasible to consider that the following IADs were stable: unruptured, small, fusiform, not flow-related, and distal (12–15). For these spontaneous lesions, close follow-up and conservative medication can be recommended first.

In 2011, Mizutani et al. summarized four clinical and pathological courses of IADs: (a) occurrence and healing with no manifestation, (b) occurrence with headache and healing with no manifestation, (c) occurrence and subsequent infarction, and (d) occurrence and subsequent SAH (9). The first and second courses may account for most cases. The third course can occur in the MCA and basilar artery (BA). The fourth course occurs in the posterior circulation more commonly (16, 17). Ruptured IADs can have high mortality under medical treatment only, with a rate of approximately 8.3% (18). The rebleeding rate is high at 55% (18–75%), and rebleeding usually occurs within 24 h of the initial rupture (7, 19).

## 4 Angiography and magnetic resonance of IADs

### 4.1 Angiography

**Figure 2:**
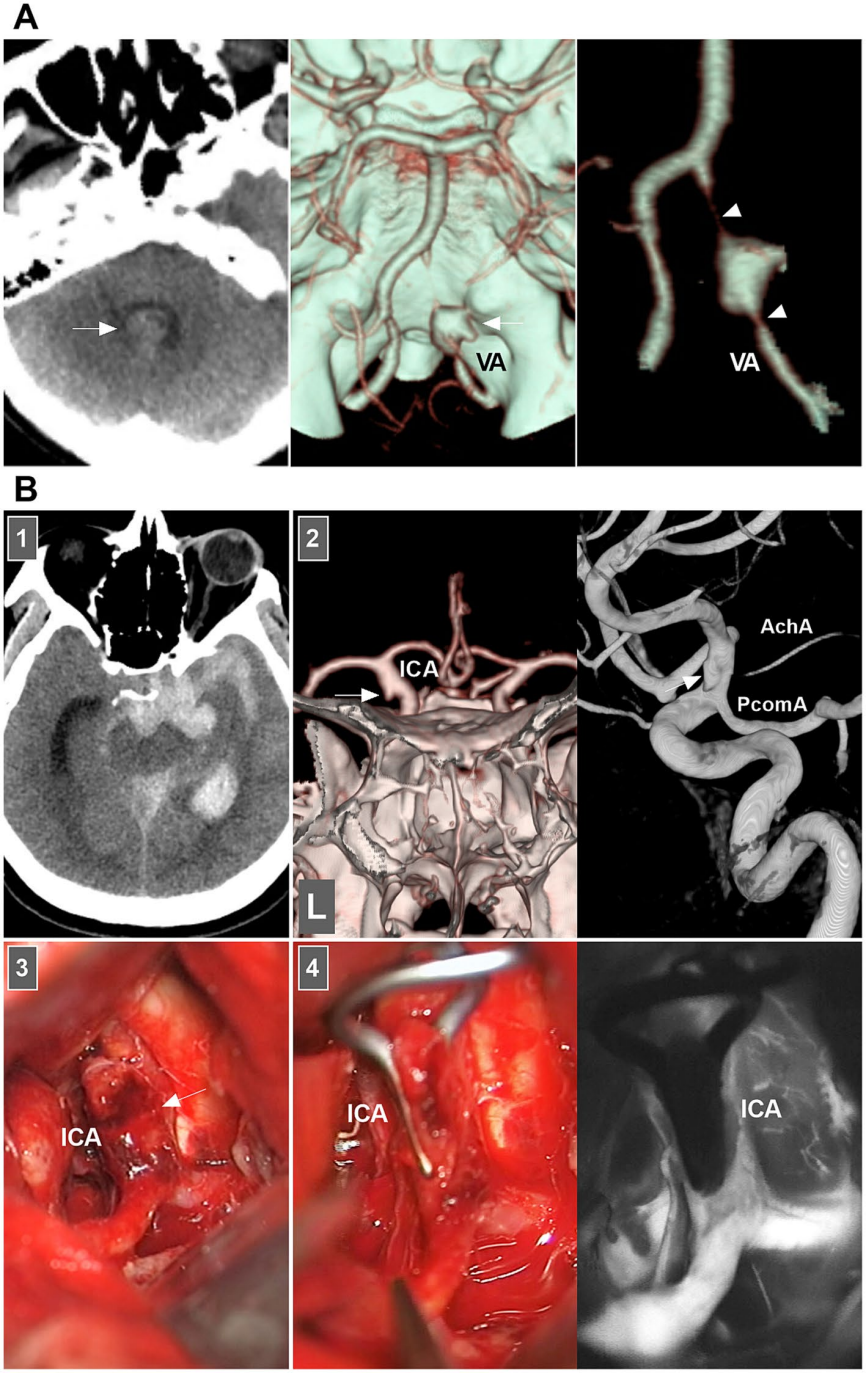
IAD appearance on angiography and open surgery. **(A)** Left panel: CT showing the hemorrhage in the fourth ventricle (arrow). Middle and right panels: CT images showing a typical VA dissection (arrow in middle panel) presenting with dilatation with stenosis (arrowheads in right panel). **(B)** Panel 1: CT showing subarachnoid hemorrhage into temporal horn of lateral ventricle. Panel 2: CTA (left panel) and DSA (right panel) images showing a protrusion (arrows) on the left supraclinoid ICA above the PcomA and opposite to the AchA, indicating that the lesion was a dissection or blood blister-like aneurysm. Panel 3: Intraoperative image showing that supraclinoid ICA had subadventitia hemorrhage (arrow), confirming to the dissection not a blood blister-like aneurysm. Panel 4: Intraoperative image (left panel) showing the clipping was performed, intraoperative fluoroscopy (right panel) showing that intracranial ICA was patent. AchA, anterior choroidal artery; CT, computed tomography; CTA, CT angiography; DSA, digital subtraction angiography; IAD, intracranial arterial dissection; ICA, internal carotid artery; PcomA, posterior communicating artery; VA, vertebral artery.

When both the entry and exit can be visualized via angiography, a double lumen is considered (1). When IADs extend inward with no exit on angiography, narrowing or occlusion of the vessel should be considered. When the media penetrates the subadventitia on angiography, aneurysmal dilatation of the outer wall of the vessel should be considered. When the adventitia has penetrated, SAH is likely (Figure 2A) (20–22).

Therefore, according to the European Stroke Organization (ESO) guidelines and a review in Lancet Neurology (2015), IADs are confirmed on the basis of at least one of the following: (a) angiography reveals stenosis or occlusion of an intracranial artery secondarily developing toward a fusiform or irregular aneurysmal dilation at a nonbranching site; (b) angiography revealing an intramural hematoma, intimal flap, or double lumen; and (c) pathological examination confirming the IAD (Figure 2B) (1, 7).

### 4.2 Magnetic resonance

Vessel wall magnetic resonance (MR) has emerged as pivotal imaging for diagnosing IADs. On high-resolution three-dimensional pre- and post-Gd black blood T1WI images and T2WI images from a 3 Tesla MR machine, direct critical features for dissection can be found, such as the intimal flap, double lumen, intramural hematoma, and abnormal arterial wall thickening and enhancement (23). The following MR images are useful tools for the diagnosis of IADs: susceptibility-weighted images, three-dimensional simultaneous noncontrast angiography, intraplaque hemorrhage, three-dimensional phase-sensitive inversion recovery, three-dimensional improved motion-sensitized driven equilibrium preparation, proton-density weighted images, high-resolution compressed-sensing time-of-flight MR angiography, and noncontracting three-dimensional time-of-flight magnetic resonance angiography (24–29). High-resolution vessel wall MR wall enhancement has been reported to be useful in predicting the rupture point of dissecting aneurysms (30). In addition, wall enhancement after EVT can predict the progression and delayed rupture of IADs (31).

## 5 EVT indications for IADs

In 2021, ESO released a specific guideline for the management of dissections. It is the first attempt at a comprehensive guideline dedicated to arterial dissections (7). Furthermore, in 2022, the American Stroke Association (ASA) proposed more specific guidelines amid uncertainty about the ESO guidelines (32).

### 5.1 ESO guidelines

For ruptured IADs with SAH, early intervention is recommended, and various EVT techniques can be used. For unruptured IADs with acute ischemia, intravenous thrombolysis is suggested. For unruptured IADs with acute large artery occlusion, EVT within 4.5 h of onset is suggested. For unruptured IAD patients with acute ischemia or TIA, antiplatelet agents may have a better risk/benefit ratio than anticoagulants do. For unruptured IAD patients with an intracranial dissecting aneurysm and isolated headache, the benefits and risks of EVT or surgical treatment are unclear unless the size of the aneurysm has increased significantly on follow-up imaging or if there are signs of compression (7).

### 5.2 ASA guidelines

According to the ASA acute stroke guidelines, the benefits of thrombolytics such as recombinant tissue-type plasminogen activators in patients with IADs are unclear. According to the ASA secondary prevention guidelines, rescue EVT techniques may be used for IADs with recurrent or progressive symptoms. It is necessary to perform multidisciplinary assessments to ascertain the best therapeutic approaches for IADs (32).

### 5.3 Other suggestions

Other suggestions included the safety and effect of intravenous thrombolysis, antiplatelet therapy and EVT on IADs.

In 2025, on the basis of a retrospective matched-pair cohort study that used a nationwide inpatient database in Japan, Egashira et al. (33) performed a study that enrolled 242 patients, and the safety and outcomes of intravenous thrombolysis in acute ischemic stroke patients with IAD were assessed. This study revealed that patients with underlying IAD may face an increased risk of intracranial hemorrhage and a reduced chance of functional recovery following intravenous thrombolysis compared with those without IAD. This study indicated that intravenous thrombolysis was not suggested for managing acute ischemic stroke with IAD.

However, antiplatelet therapy may be useful for treating ischemic IADs, and EVT may be used in select patients with IAD. For example, in 2023, Shimizu et al. (34) performed a Japanese nationwide survey of treatments for IAD causing cerebral ischemia within 2 weeks at 35 neurological centers, and the results revealed that patients with intracranial carotid dissection causing cerebral ischemia who underwent stenotic dissection were at risk of further aggravation and that EVT could improve or prevent aggravation. In 2019, Al-Mufti et al. (35) performed a systematic review of 82 studies, including a total of 669 patients with anterior circulation IADs [492 (74%) with ischemia] and 2,948 patients with posterior circulation IADs [960 (33%) with ischemia]. In this review, researchers suggested antiplatelet therapy for patients with ischemic IADs and considered EVT for patients with SAH.

However, these studies were retrospective, the evidence level was low, and randomized controlled trials are necessary.

## 6 EVT techniques to treat IADs

**Figure 3:**
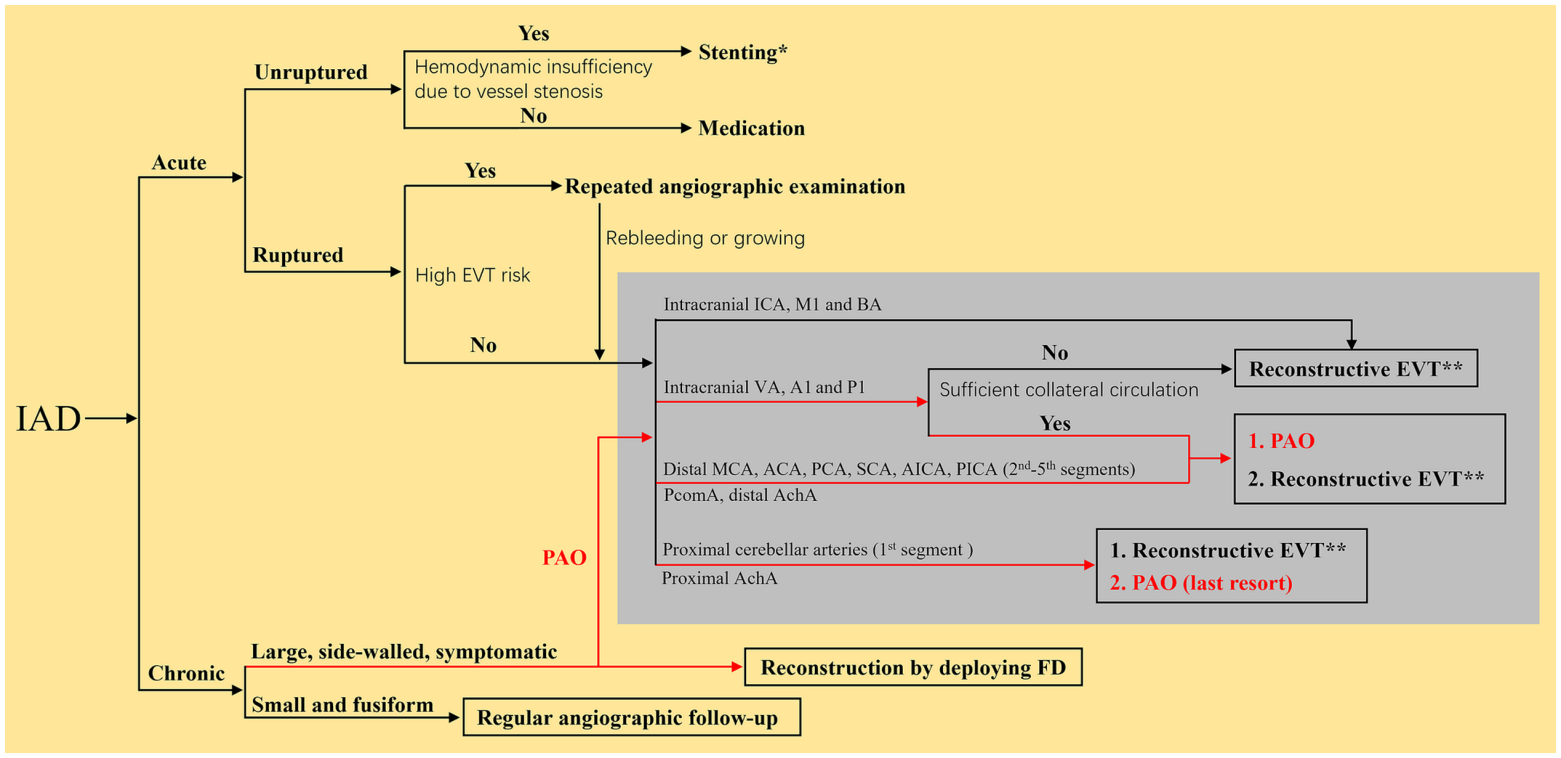
Flow chart of the EVT choice for IADs. ^*^Indicated that the reconstruction EVT was recommended to use traditional self-expanding stents with low-metal coverage rate. ^**^Indicated that the reconstructive EVT was recommended to use braided stents priorly. The red roads mean that the PAO can also be an EVT choice for chronic IADs. ACA, anterior cerebral artery; AchA, anterior choroidal artery; A1, first segment of ACA; BA, basilar artery; EVT, endovascular treatment; FD, flow diverter; IAD, intracranial arterial dissection; ICA, internal carotid artery; MCA, middle cerebral artery; M1, first segment of MCA; PCA, posterior cerebral artery; P1, first segment of PCA; SCA, superior cerebellar artery; AICA, anterior inferior cerebellar artery; PAO, parent artery occlusion; PcomA, posterior communicating artery; PICA, posterior inferior cerebellar artery; VA, vertebral artery.

The optimal EVT strategy for IADs remains unclear. In the absence of randomized controlled trials and considering the limited data from observational studies with a high risk of bias, all experts recommend selecting the optimal intervention on the basis of a multidisciplinary assessment (7). The elimination of IADs and reconstruction of the parent artery must be the prime objective (36, 37). The flowchart of the EVT choice for IADs is shown in Figure 3, which can provide some suggestions for doctors.

### 6.1 Ischemic IAD

Angioplasty or stenting to restore blood flow is useful. Reconstructive EVT should use stents with good radial force to improve dissecting healing (10). In 2015, Kim et al. (38) evaluated the efficacy of a self-expanding stent for ischemic anterior circulation IADs and confirmed a positive outcome for lesions presenting with acute/crescendo-type stroke or recurrent ischemia despite adequate medication. In addition, mechanical thrombectomy may be used to recanalize occluded large vessels (39).

### 6.2 Hemorrhagic, symptomatic or progressive IADs

Four types of EVT techniques can be used to treat these IADs: (a) isolation of the IAD to exclude blood flow from the dissected region, (b) parent artery occlusion (PAO) at the proximal IAD to reduce blood flow in the dissected region, (c) isolation of both the IAD and PAO, and (d) occlusion or isolation of aneurysmal dilatation by stenting with/without coiling but preservation of the parent artery (1). Options 1, 2 and 3 are reconstructive EVT techniques; option 4 is a reconstructive EVT technique.

When performing deconstructive EVT, the collateral should be assessed with a balloon-occlusion test (BOT) or amobarbital infusion during angiography with simultaneous monitoring of the patient’s neurological function (1). When a patient passes the BOT, deconstructive EVT can be used. Alternatively, reconstructive EVT must be employed, including traditional stent-assisted coiling, multiple stenting, and flow diverter (FD) usage (2, 40). For hemorrhagic IADs, coiling the rupture point is necessary.

Currently, there is a growing trend in the use of FDs to reconstruct IADs (41). Braided FDs with a >30% rate of metal coverage can offer sufficient support to the arterial wall and prevent further IAD progression by acting as scaffolding, redirecting blood flow and inducing thrombosis. The new generation of FDs with surface modifications may reduce the use of antiplatelet therapy, which may be promising (42).

## 7 Prognosis and complications of EVT for IADs

For patients with IADs treated by EVT, the clinical outcome can be assessed via the modified Rankin Scale (mRS), and an mRS score of 0–2 is considered a good outcome (17). On angiography, adequate aneurysm occlusion was defined as complete occlusion or near complete occlusion with a small residual neck. For FD deployment, adequate aneurysm occlusion was defined as O’Kelly Marotta grade C or D (41).

### 7.1 Prognosis

According to previous reports, deconstructive and reconstructive EVT for IADs can result in good clinical outcomes and adequate aneurysm occlusion (Table 1). Deconstructive EVT may lead to a higher complete aneurysm occlusion rate; however, reconstructive EVT may lead to a higher rate of good clinical outcomes (43). Reconstructive EVT by traditional stenting and FD deployment are effective in treating IADs (44). However, for large and complex IADs, FD deployment can be a promising approach to reduce IAD recurrence (45). For example, in Amoukhteh’s et al. (41) meta-analysis, after FD deployment for IADs, at the last follow-up, the aneurysm recurrence/rebleeding rate was only 0.1%.

**Table 1:** EVT outcomes for IADs in a recent systematic review and meta-analysis.

Author, year	Outcomes
Prestes et al., 2024 (2)	In 17 studies, comparing 173 solo stenting and 377 stent-assisted coiling procedures for posterior circulation IADs, the findings suggest there is no substantial basis for favoring stent-assisted coiling over solo stenting across all cases
Amoukhteh et al., 2024 (41)	In 20 studies with 329 patients, FD in the treatment of IADs revealed an 89.7% rate of favorable clinical outcome, an adequate occlusion rate of 88.3% and a mortality rate of 2.4%. The aneurysm recurrence/rebleeding rate was 0.1%, in-stent stenosis/thrombosis occurred at a rate of 1.14%, and ischemic events/infarctions were seen in 3.3% of cases. The need for retreatment was 0.9%, and the technical success rate was impressively high at 99.1%
Essibayi et al., 2024 (43)	In 56 studies with 1,095 cases with intracranial VA and ICA dissections, deconstructive EVT was applied in 40.9% cases and had a rate of complete aneurysm occlusion of 86.4%, a rate of a good clinical outcomes of 72.1% and a mortality rate of 15.1%, compared to 70.2, 83.3, and 9.5%, respectively, for reconstructive EVT. For reconstructive EVT, procedural complication was in 12.6% patients. For deconstructive EVT, procedural complication was in 16.9% patients
Amoukhteh et al., 2024 (44)	In 6 studies involving 131 patients in the FD group and 199 patients in the traditional stent group, the rates of favorable functional outcomes (86.8% vs. 86%), mortality (3.9% vs. 6%), adequate aneurysms occlusion (79.7% vs. 86.3%), aneurysm recurrence (1.3% vs. 13.3%), in-stent stenosis/thrombosis (7% vs. 6.9%), ischemic events/infarctions (6.7% vs. 7.8%), retreatment (7% vs. 8.6%), and technical success (100% vs. 98.7%) were comparable in individuals treated with FD and traditional stent
Brenner et al., 2024 (45)	In 10 studies, 195 and 222 patients were included in the FD and the stent-assisted coiling group, and both techniques achieved similar postoperative complete aneurysmal occlusion rates in angiographic follow-up. The techniques had similar complication rates

EVT, endovascular treatment; FD, flow diverter; IAD, intracranial arterial dissection; ICA, internal carotid artery; VA, vertebral artery.

### 7.2 Complications

For EVT for IADs, complications are unavoidable, including hemorrhagic or ischemic types, such as intraoperative rupture of the IAD, perforator occlusion, in-stent thrombosis, and postoperative ischemia due to hemodynamic alterations from PAO. In Essibayi’s et al. (43) meta-analysis, procedure-related complications were reported in 12.6% of patients who underwent reconstructive EVT and 16.9% of patients who underwent deconstructive EVT, and there was no difference between these two approaches.

## 8 EVT techniques for each type of IAD

### 8.1 Anterior circulation IADs

#### 8.1.1 IADs of the supraclinoid ICA, posterior communicating artery and anterior choroidal artery

Supraclinoid ICA dissections are infrequent and can be divided into chronic and acute types (46). Chronic lesions are slow growing and often have a large and fusiform dilated shape. Acute lesions often rupture suddenly, causing SAH or ICA stenosis or occlusion, which can cause cerebral ischemia. Supraclinoid ICA dissections are difficult to manage via deconstructive EVT because of the need to preserve the anterior choroidal artery (AchA) or posterior communicating artery (PcomA) (11).

**Figure 4:**
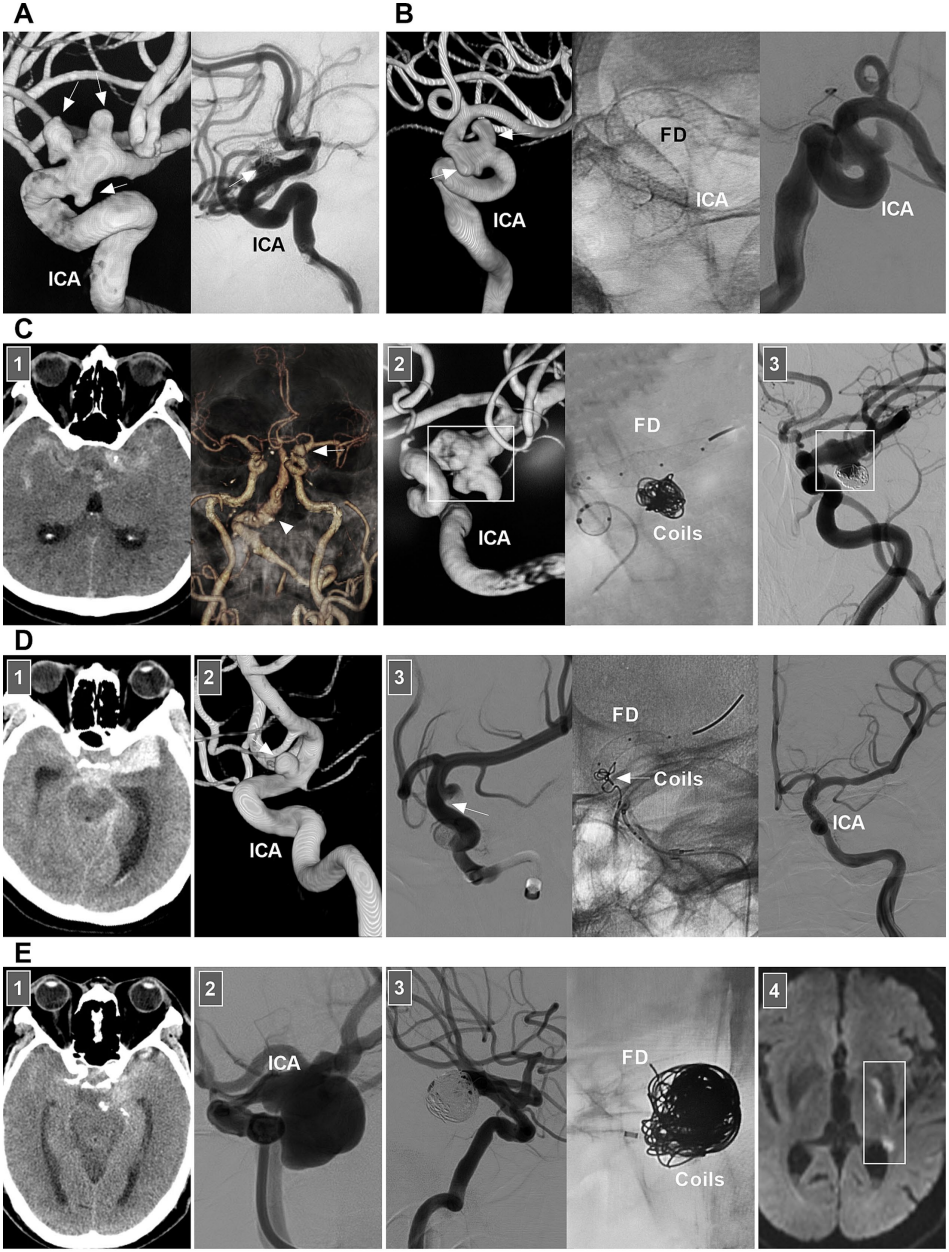
EVT of supraclinoid ICA dissections. **(A)** Left panel: DSA showing multiple dissecting aneurysms (arrows) on supraclinoid ICA. Right panel: one-year follow-up DSA showing the embolization was incomplete after traditional stent-assisted coiling (arrow). **(B)** Left panel: DSA showing multiple dissecting aneurysms (arrows) on supraclinoid ICA. Middle panel: X-ray image showing the FD in supraclinoid ICA. Right panel: DSA showing the ICA reconstruction. **(C)** Panel 1: CT (left panel) showing SAH; CTA (right panel) showing a PcomA aneurysm (arrow) and vertebrobasilar dilatation (arrowhead). Panel 2: DSA (left panel) showing a supraclinoid ICA dissection with a PcomA aneurysm (frame); X-ray image (right panel) showing FD deployment with coiling to treat the dissection. Panel 3: Six-month follow-up DSA showing that the supraclinoid ICA was reconstructed (frame). **(D)** Panel 1: CT showing SAH. Panel 2: DSA showing a supraclinoid ICA aneurysm (arrow). Panel 3: DSA (left panel) showing that the aneurysm was dissecting, the arrow indicated the entry of the dissection; X-ray image (middle panel) showing the entry of the dissection was coiled (arrow) under the assistance of FD deployment; DSA (right panel) showing the dissection cannot be seen. **(E)** Panel 1: CT showing SAH. Panel 2: DSA showing a large supraclinoid ICA dissecting aneurysm (arrow). Panel 3: DSA (left panel) showing that the aneurysm was coiled, X-ray image (right panel) showing the coiling under the assistance of FD deployment. Panel 4: Diffuse weighted image of magnetic resonance showing acute ischemia (frame) of the region supplied by anterior choroidal artery. CT, computed tomography; CTA, CT angiography; DSA, digital subtraction angiography; EVT, endovascular treatment; FD, flow diverter; ICA, internal carotid artery; PcomA, posterior communicating artery; SAH, subarachnoid hemorrhage.

For chronic unruptured dissections, FD deployment without/with coiling is more effective than traditional stent-assisted coiling is (Figures 4A,B). For chronic ICA dissections with a ruptured bleb, selective coiling of the ruptured point is necessary (Figure 4C) (47). For acute stenotic ICA dissections with progressive neurologic deficits due to cerebral hyperperfusion syndrome, the use of stenting to reconstruct the ICA and close the entry point of the dissection is needed. According to previous reports, traditional self-expanding stenting is effective (38, 48, 49). If EVT fails or cannot be performed, extracranial–intracranial bypass must be the last resort (34, 50). For ruptured acute ICA dissections with aneurysm formation, traditional stenting may be insufficient, and FD deployment with coiling rupture points is a good choice (Figure 4D). However, in acute SAH, FD deployment to reconstruct the ICA may be associated with ischemic complications, which should be considered (Figure 4E).

**Figure 5:**
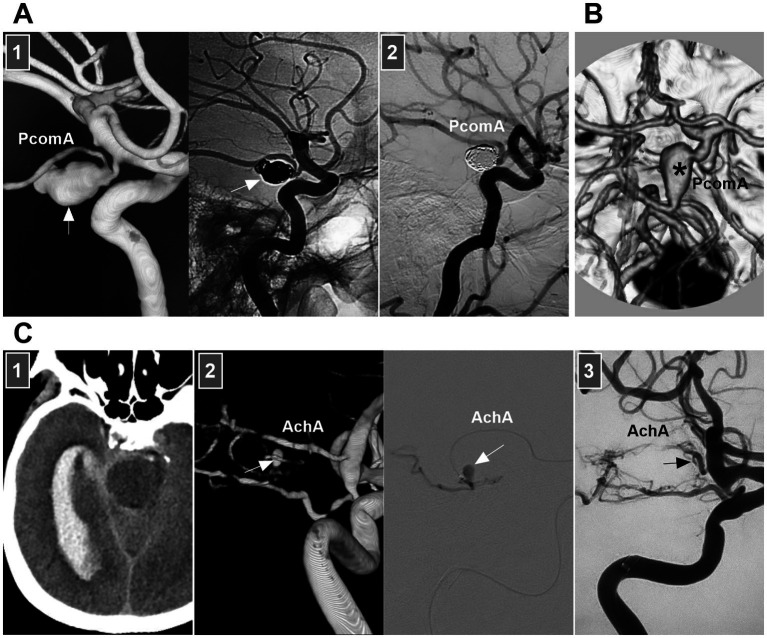
EVT of PcomA and AchA dissections. **(A)** Panel 1: DSA (left panel) showing a PcomA trunk dissecting aneurysm (arrow), unsubtracted DSA (right panel) showing that the aneurysm was coiled (arrow), the PcomA was preserved. Panel 2: Four-year follow-up DSA showing the aneurysm had no recurrence. **(B)** CTA showing a PcomA trunk dissecting aneurysm (asterisk), the lesion can be treated by parent artery occlusion. **(C)** Panel 1: CT showing subarachnoid hemorrhage. Panel 2: DSA (left panel) and microcatheter angiography (right panel) showing a dissecting aneurysm (arrow) at distal AchA. Panel 3: DSA showing that the dissection was occluded by casting liquid embolic agent and the proximal AchA trunk (arrow) was preserved. AchA, anterior choroidal artery; CT, computed tomography; CTA, CT angiography; DSA, digital subtraction angiography; EVT, endovascular treatment; PcomA, posterior communicating artery.

As branches of the supraclinoid ICA, rarely, the AchA and PcomA trunks can undergo spontaneous dissections, and PcomA trunk dissection can be performed selectively or completely by coiling (Figures 5A,B) (51). AchA trunk dissections are often flow related to arteriovenous malformation (AVM) and moyamoya disease. For distal AchA dissection, deconstructive EVT via a liquid embolic agent can be used (Figure 5C). For proximal AchA dissection, because of its thin diameter, stenting is difficult, and deconstructive EVT has to be the last resort.

#### 8.1.2 MCA dissection

For acute unruptured MCA dissections, even with aneurysm formation, antithrombotic treatment should be prioritized (52, 53). Thrombectomy with/without stenting can be attempted only for acute occluded dissection (54). EVT can be recommended for hemorrhage and confirmed dissecting aneurysms (55). M1 occlusion or isolation of the dissection without efficient bypass poses a significant risk of MCA territory infarct. Although bypass to the distal MCA is often successful, lenticulostriate artery infarction is often inevitable (55). Therefore, preventing rupture and stabilizing the dissected wall while preserving arterial continuity seems to be optimal, allowing subsequent healing via endothelialization.

**Figure 6:**
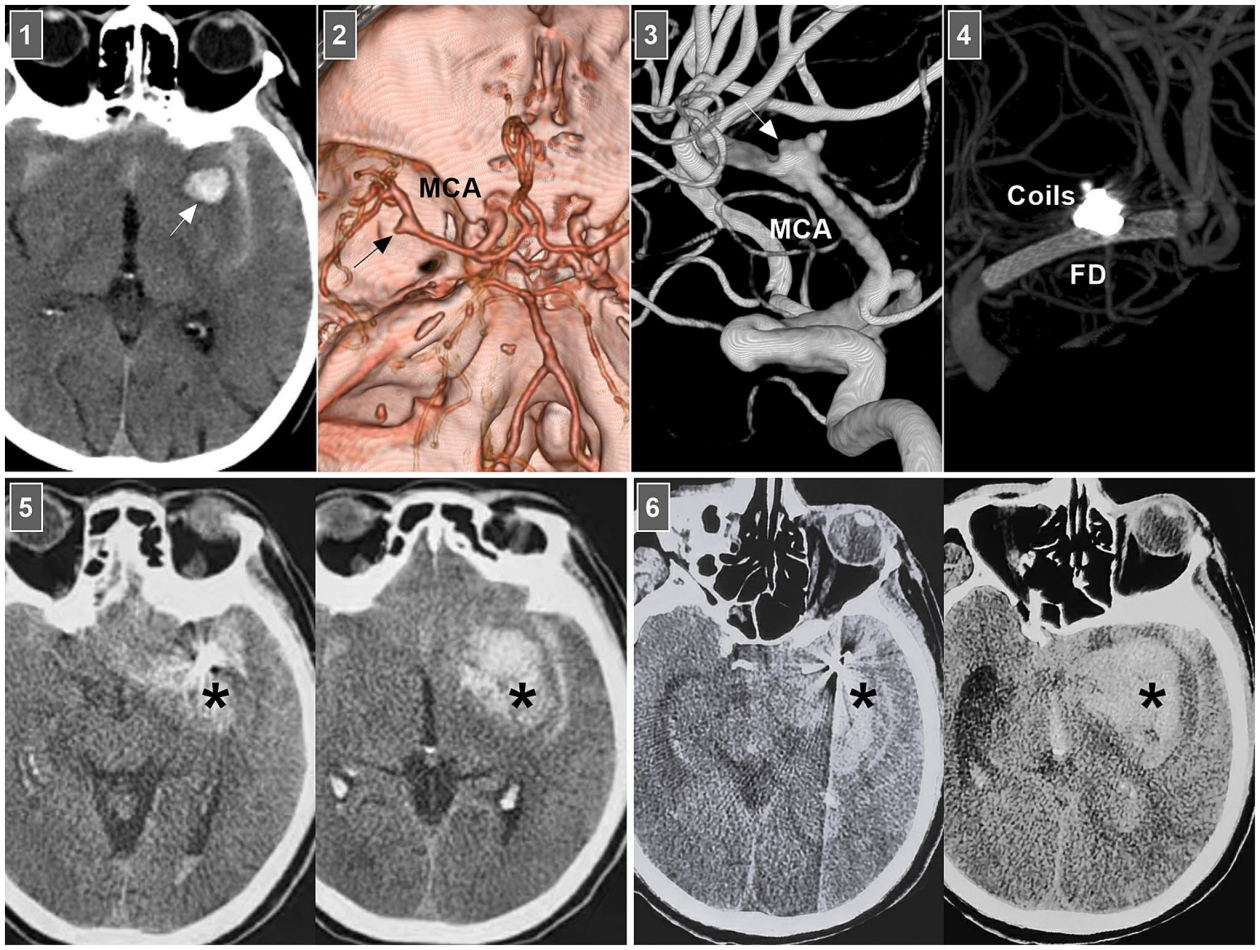
Rebleeding after EVT of an MCA dissection. Panel 1: CT showing subarachnoid hemorrhage and parenchymal hematoma (arrow). Panel 2: CT angiography showing a left MCA trunk aneurysm (arrow). Panel 3: DSA showing that the aneurysm (arrow) was an MCA trunk dissection involving the origin of lenticulostriate artery. Panel 4: Vaso-reconstructive image showing that MCA dissection was coiled by FD assistance. Panel 5: Postoperative 1-h CT images showing increased hemorrhage (asterisks), indicating the MCA dissection reruptured. Panel 6: Postoperative 3-h CT images showing the bleeding (asterisks) continued to increase, the patient fell into coma. CT, computed tomography; DSA, digital subtraction angiography; EVT, endovascular treatment; FD, flow diverter; MCA, middle cerebral artery.

**Figure 7:**
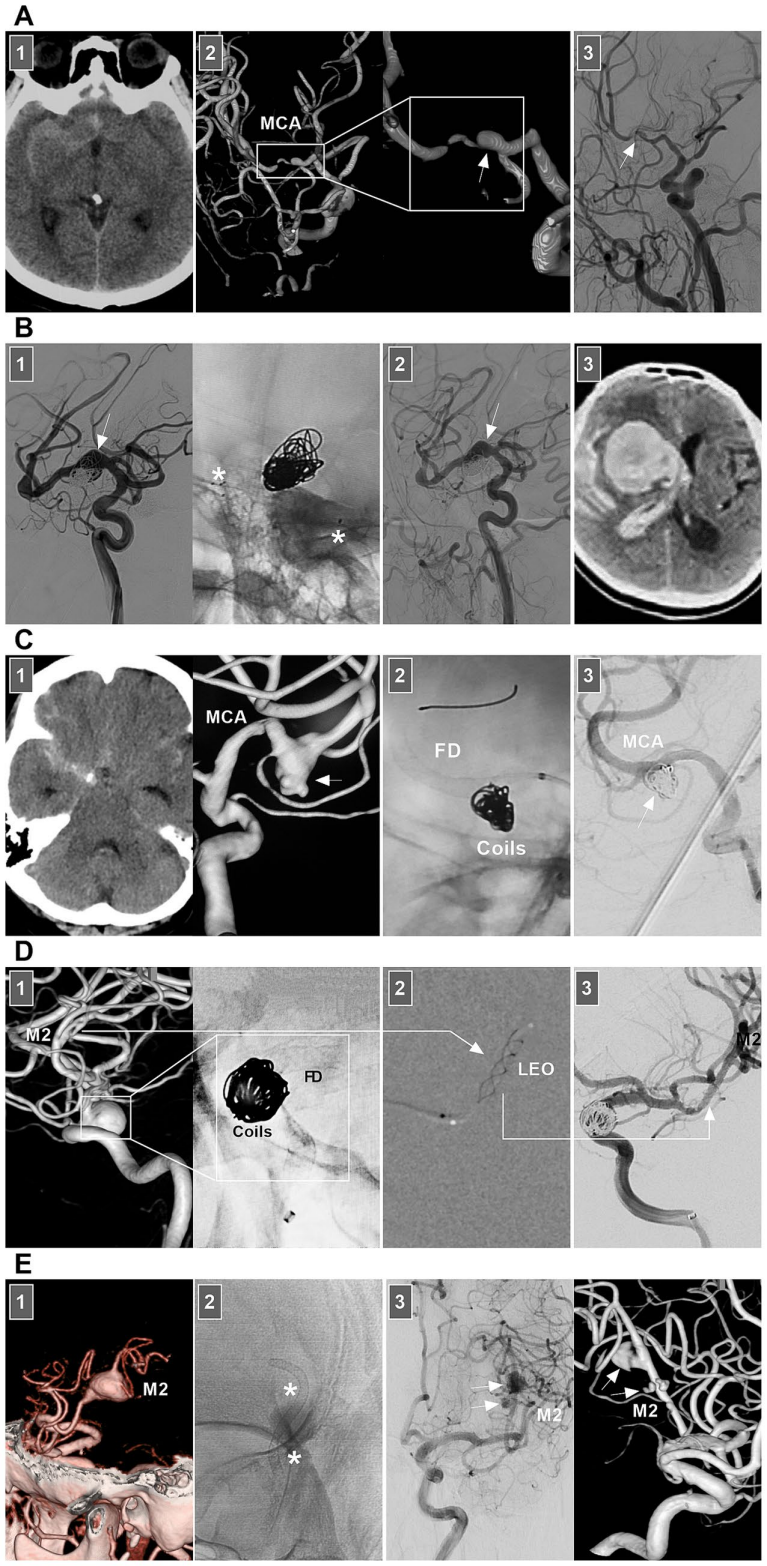
Treatment of MCA dissections. **(A)** Panel 1: CT showing SAH. Panel 2: DSA images showing a dissection (frames) at MCA trunk with stenosis and dilatation (arrow). Panel 3: Three-month follow-up DSA showing that the dissection (arrow) regressed. **(B)** Panel 1: DSA (left panel) and X-ray image (right panel) showing a fusiform MCA dissection (arrow) was coiled under the assistance of traditional stenting (asterisks). Panel 2: One-year follow-up DSA showing that the dissecting aneurysm (arrow) was stable and did not grow. Panel 3: Three-year follow-up CT showing fatal intracranial hemorrhage, indicating the dissection ruptured. **(C)** Panel 1: CT (left panel) showing SAH, DSA (right panel) showing an MCA dissecting aneurysm (arrow). Panel 2: X-ray image showing the FD-assisted coiling. Panel 3: DSA showing that the dissecting aneurysm (arrow) was embolized. **(D)** Panel 1: DSA (left panel) and X-ray image (right panel) showing a supraclinoid ICA aneurysm was coiled by the assistance of FD deployment (frames), there was a M2 dissection. Panel 2: Roadmap image showing the M2 dissection was stented by LEO baby (long arrow). Panel 3: DSA showing the reconstructed M2 (long arrow). **(E)** Panel 1: CT angiography showing a M2 dissection. Panel 2: X-ray image showing the dissection was stented by FD deployment (asterisks). Panel 3: DSA (left panel) and its reconstructive image (right panel) of six-month follow-up showing that the M2 segment was reconstructed with less residual dissection (arrows). CT, computed tomography; DSA, digital subtraction angiography; EVT, endovascular treatment; FD, flow diverter; MCA, middle cerebral artery; M2, second segment of MCA; SAH, subarachnoid hemorrhage.

In selective hemorrhagic MCA dissection without severe stenosis, stent-assisted coiling may be effective (55, 56). For acute dissection, the rupture site is very fragile, and excessive coil packing should be avoided due to the risk of rupture (Figure 6). FDs can decrease blood flow into the rupture site. However, for stenotic MCAs, delivering the FD through a thick microcatheter is often difficult. For hemorrhagic MCA dissection with severe stenosis, conservative treatment had to be the last resort (Figure 7A).

**Figure 8:**
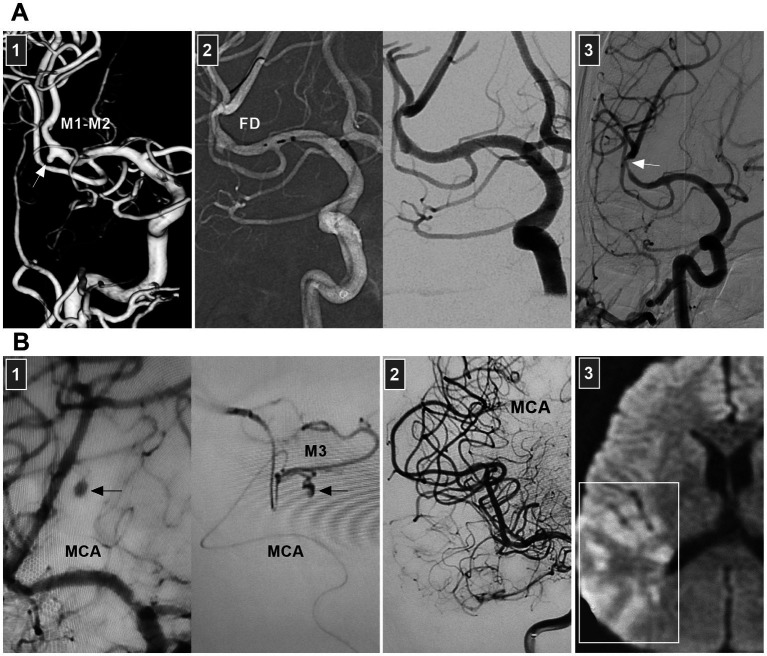
Complication from EVT for MCA dissections. **(A)** Panel 1: DSA showing a sidewall dissection at M1–M2 junction (arrow). Panel 2: Roadmap image (left panel) and DSA (right panel) showing the dissection was covered by FD. Panel 3: Six-month follow-up DSA showing that the dissection was cured and there was a stenosis of M2 (arrow). **(B)** Panel 1: DSA (left panel) and microcatheter angiography (right panel) showing a small dissection of M3 (arrows). Panel 2: DSA showing the dissection was embolized by casting liquid embolic agent. Panel 3: Postoperative magnetic resonance image showing that the acute infarction of parietal lobe (frame). DSA, digital subtraction angiography; EVT, endovascular treatment; FD, flow diverter; MCA, middle cerebral artery; M1, first segment of MCA; M2, second segment of MCA; M3, third segment of MCA.

Chronic MCA dissections can present with fusiform or sidewall aneurysmal dilatation. For M1 dissections, FD deployment to reconstruct the M1 segment is an option (Figures 7B,C). For M2 dissections, reconstructive EVT is recommended (Figures 7D,E). However, complications associated with FD deployment must be considered (Figures 6, 8A). If the dissections of the M2 segment of the inferior trunk are giant with thrombi, the distal MCA may experience ischemic preconditioning, and deconstructive EVT is acceptable (57). PAO for M3–M4 dissections can be performed because of adequate leptomeningeal and pial collaterals from the anterior cerebral artery (ACA) and posterior cerebral artery (PCA) (57). In addition, for flow-related distal MCA dissections, deconstructive EVT can be aggressively performed. However, if branches are supplied to important functional areas, such as the central sulcal artery, precentral sulcal artery and postcentral sulcal artery, PAO should be performed cautiously (Figure 8B) (58).

#### 8.1.3 ACA dissection

**Figure 9:**
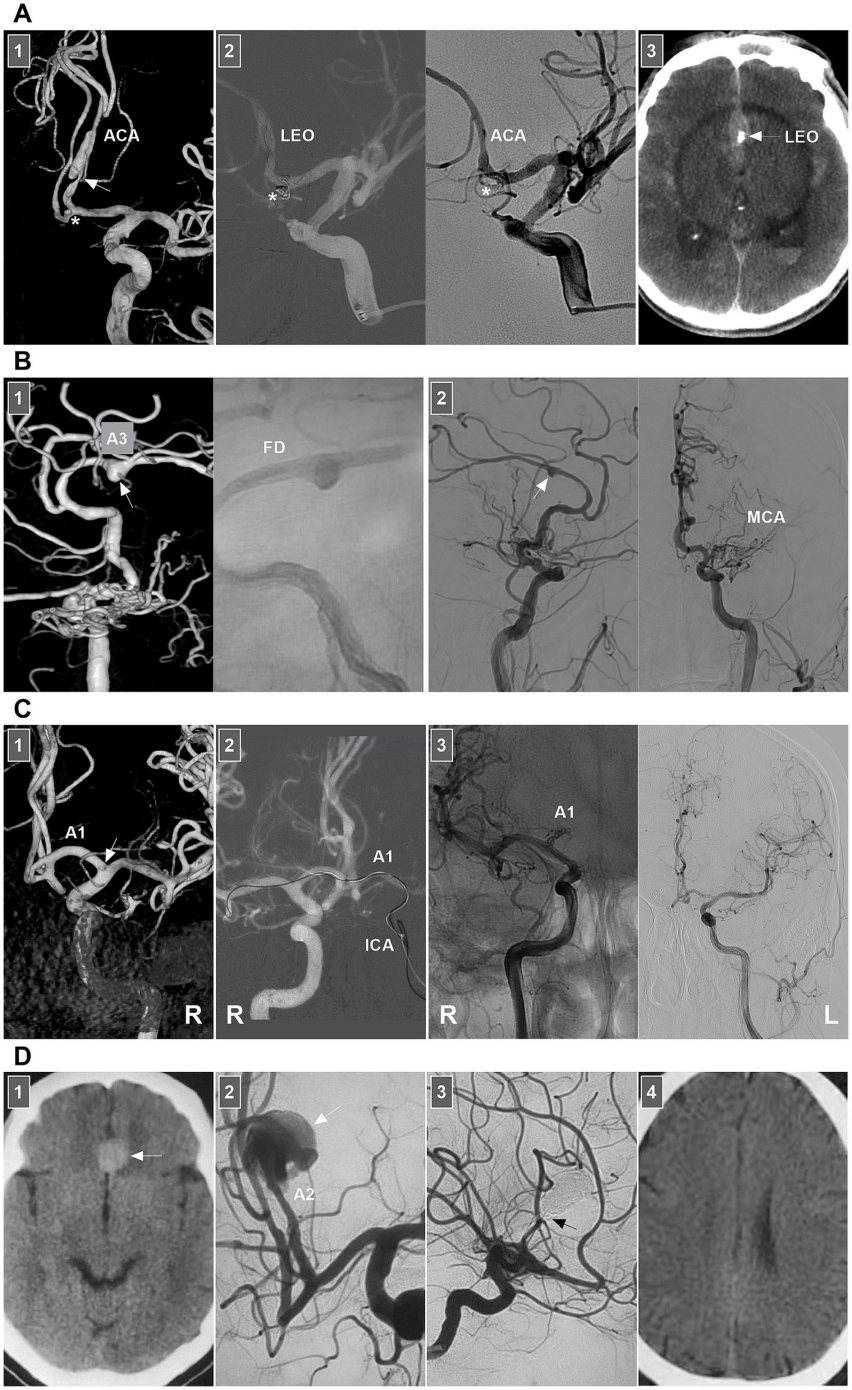
EVT of ACA dissections. **(A)** Panel 1: DSA showing a typical ruptured dissection (arrow) at A2 segment trunk and an AcomA aneurysm (asterisk). Panel 2: Roadmap image (left panel) and DSA (right panel) showing that the AcomA aneurysm was coiled (asterisks) and the dissection was reconstructed by LEO baby stenting. Panel 3: Postoperative X-per CT showing the LEO stent (arrow) was in the hematoma of anterior longitudinal fissure, indicating the ACA dissection was ruptured. **(B)** Panel 1: DSA (left panel) showing a fusiform A3 dissection (arrow), X-ray image (right panel) showing that the A3 dissection was covered by FD. Panel 2: Four-month follow-up DSA (left panel) showing the A3 dissection regressed, DSA showing the previous MCA occlusion. **(C)** Panel 1: DSA showing a small dissection at right A1 origin (arrow). Panel 2: Roadmap image showing that the contralateral trans-circulation approach to treat the dissection by occluding A1 origin. Panel 3: Six-month follow-up DSA (left and right panels) showing that right A1 origin was occluded, left ACA supplied the bilateral ACAs. **(D)** Panel 1: CT showing a lesion (arrow) in the anterior longitudinal fissure. Panel 2: DSA showing a giant A2 dissecting aneurysm (arrow). Panel 3: DSA showing the aneurysms was trapped and the proximal A2 was occluded (arrow). Panel 4: Postoperative CT showing no ischemic finding in the frontal lobe. ACA, anterior cerebral artery; A1, first segment of the ACA; A2, second segment of the ACA; A3, third segment of the ACA; AcomA, anterior communicating artery; CT, computed tomography; DSA, digital subtraction angiography; EVT, endovascular treatment; FD, flow diverter; ICA, internal carotid artery; L, left; R, right.

ACA dissections are rare. Most ischemic dissections occur at the A2 segment (59, 60). However, hemorrhagic dissections can occur at any segment of the ACA (60, 61). ACA dissections with only ischemic onset can usually be successfully treated conservatively, although there are reports that stenting successfully treats these ischemic lesions (62, 63). For ruptured dissections, if there is a high risk of rebleeding under conservative treatment or if unruptured symptomatic ACA dissections grow progressively, EVT can be proposed. For ruptured stenotic ACA dissections, it may be sufficient to close the entry of the dissection by stenting (Figure 9A). For sidewall dissections, coiling with/without assistance from traditional stents may be sufficient (64). However, for chronic unruptured large fusiform ACA dissections, FDs alone or with coiling can be more effective (Figure 9B) (65).

EVT to reconstruct the ACA is the prime objective. However, when reconstructive EVT is difficult, deconstructive EVT must be used (66). For A1 dissections, when there is a competent anterior communicating artery, PAO and aneurysm isolation can be performed (Figure 9C) (64). For A2 dissections, when bypass of the ipsilateral A2 from the contralateral A2 or extracranial arteries cannot be performed, deconstructive EVT can be the last resort (Figure 9D). For A3–A5 dissections, deconstructive EVT can be considered (67–69). For flow-related dissections, deconstructive EVT can be aggressive (70).

### 8.2 Posterior circulation IADs

#### 8.2.1 BA dissections

**Figure 10:**
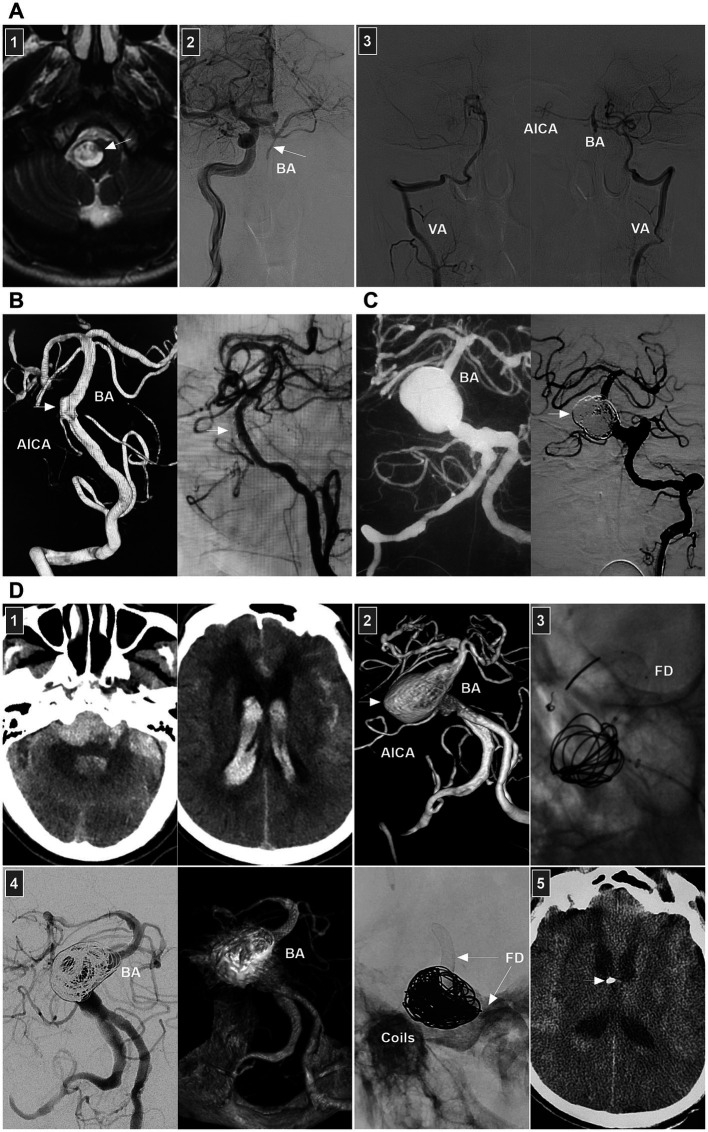
Treatment of BA dissections. **(A)** Panel 1: Magnetic resonance image showing a lesion (arrow) in front of brainstem. Panel 2: DSA showing the BA was occluded (arrow), the BA tip was supplied by posterior communicating artery. Panel 3: DSA images showing that bilateral VAs only supplied to the PICAs, lower BA and AICA. In the patient, the medication was given. **(B)** Left panel: DSA showing an unruptured BA dissection (arrow). Right panel: DSA showing delayed appearance of BA dissection after LEO stent deployment. **(C)** Left panel: Roadmap image showing a ruptured sidewall BA dissecting aneurysm. Right panel: DSA showing the aneurysm (arrow) was coiled under the assistance of traditional stent. **(D)** Panel 1: CT images (left and right panels) showing subarachnoid hemorrhage and intraventricular hemorrhage. Panel 2: DSA showing a giant BA dissecting aneurysm (arrow). Panel 3: X-ray image showing FD assisted coiling the aneurysm. Panel 4: DSA (left panel) and Vaso-reconstructive image (right panel) showing that the aneurysm was embolized. Panel 5: X-ray image showing the FD (arrows) and coils. Panel 6: Postoperative CT showing the external ventricular drainage (arrow) was performed due to acute hydrocephalus. AICA, anterior inferior cerebellar artery; BA, basilar artery; CT, computed tomography; DSA, digital subtraction angiography; EVT, endovascular treatment; FD, flow diverter; PICA, posterior inferior cerebellar artery; VA, vertebral artery.

Little is known about the clinical manifestations of spontaneous BA dissections; these lesions may be asymptomatic and silent or may present with SAH, brainstem compression, or ischemia (71). For BA dissections with brain ischemia, conservative anticoagulation treatment is the standard approach. Chronic occluded BA dissection can have no or minor symptoms (Figure 10A) (72). However, acute occluded BA dissection may be associated with high rates of mortality and morbidity. Intervention may be necessary. After aspiration, emergency stenting to reconstruct the BA lumen can be performed (73, 74).

For ruptured BA dissection, if the treatment is considered high risk, follow-up can be considered first. When the lesion progresses, aggressive EVT can be used. Chronic BA dissections can present with sidewall, circumferential, or fusiform shapes. For chronic lesions, the optimal management method is unclear. In general, for symptomatic or progressive chronic BA dissections, after the risks and potential benefits of the intervention are balanced, EVT can be considered. Various EVT techniques, including traditional coiling, overlapping stenting, FD deployment or even PAO, can be options (75).

For small or sidewall BA dissections, traditional stenting may be feasible (Figures 10B,C). However, for large or fusiform lesions, the use of FDs seems promising (Figure 10D). Adjunctive coiling for aneurysmal dilatation can prevent rebleeding or aggravate thrombosis; however, mass effects should be considered. In addition, FD deployment can yield hemorrhagic/ischemic complications and an occupying effect. When reconstructive EVT is ineffective for giant BA dissection, flow reversal by occluding bilateral VAs or BA trunk occlusion can be applied in highly selective cases (76). For vertebrobasilar junction dissections below the BA, either FD or traditional stent-assisted coiling can be used (75). The hypoplastic VA can be occluded to avoid contralateral inflow into the dissection site.

#### 8.2.2 Intracranial VA dissections

Owing to contralateral VA compensation, even if the dissection results in intracranial VA stenosis or even occlusion, hypoperfusion syndrome is uncommon. Therefore, acute dissections are often found in patients with SAH. Ruptured lesions have a high rate of rebleeding, especially for those with “stenosis and dilation” and “lateral protrusion” (77, 78). Chronic dissections often present with fusiform or lateralized dilatation of the intracranial VA that may coexist with stenosis (79). For ruptured or unruptured intracranial VA dissections with mass effects, growth, lateral aneurysm protrusion, a size >10 mm, or symptomatic lesions, EVT may be needed. The key to EVT is preserving the posterior inferior cerebellar artery (PICA) and brainstem perforators. Reconstructive EVT is the primary goal.

**Figure 11:**
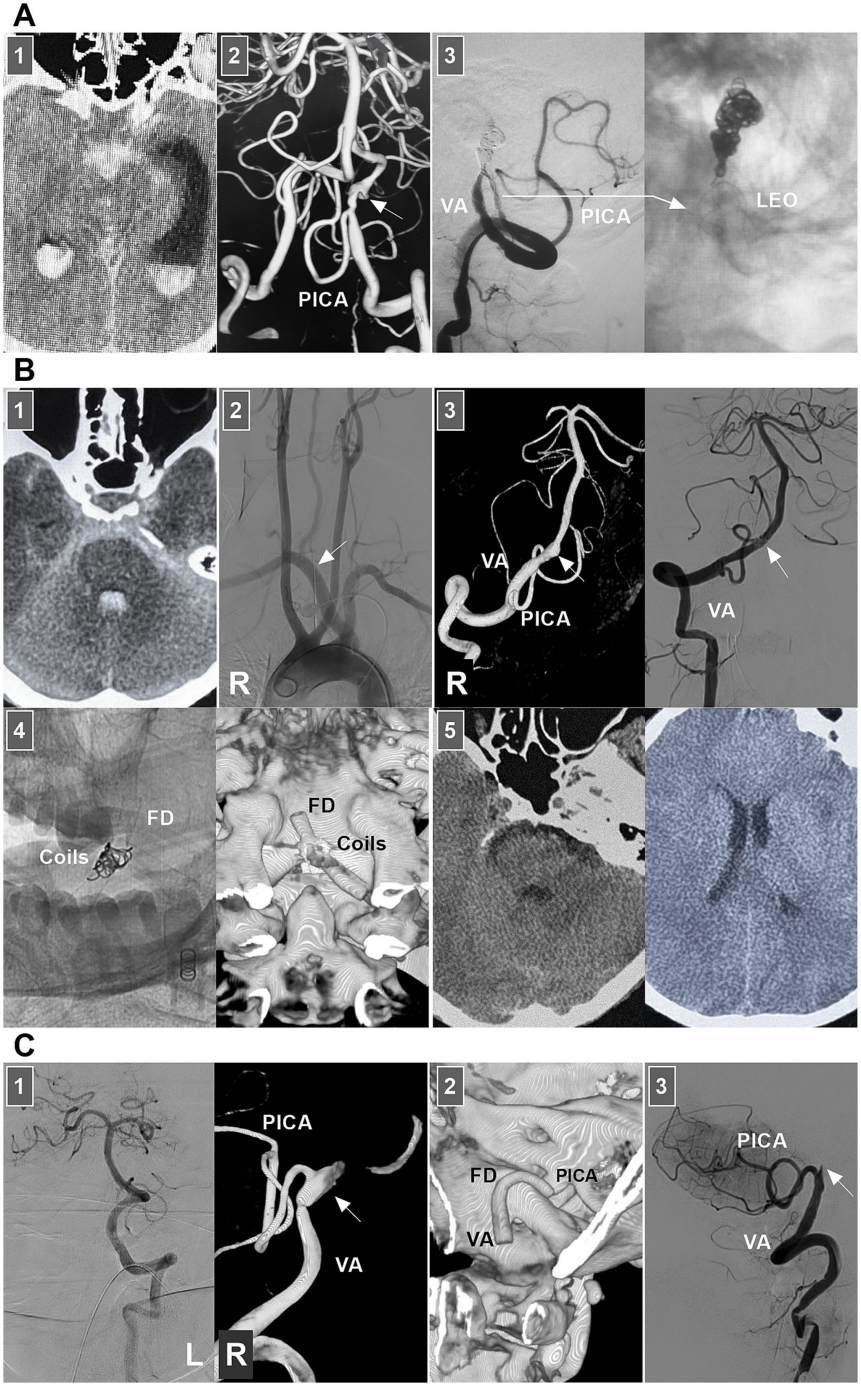
EVT of VA dissections. **(A)** Panel 1: CT showing SAH and intraventricular hemorrhage. Panel 2: DSA showing a ruptured VA dissection (arrow) above the PICA. Panel 3: DSA images (left and right panels) showing that the dissection was trapped and the PICA was stented by LEO deployment (long arrow). (B) Panel 1: CT showing SAH and intraventricular hemorrhage. Panel 2: DSA of aorta showing a right aberrant subclavian artery (arrow). Panel 3: DSA (left panel) showing a ruptured VA dissection (arrow) above the PICA, and DSA (right panel) showing the dissection (arrow) was embolized. Panel 4: X-ray image (left panel) and reconstructive Xper-CT (right panel) showing the FD and coils. Panel 5: Postoperative CT images (left and right panels) showing that intracranial hemorrhage absorbed. **(C)** Panel 1: DSA images showing that left VA was well-developed (left panel) and there was a right VA dissection (arrow) with PICA involvement (right panel). Panel 2: Reconstructive CT showing the FD deployment from PICA to VA. Panel 3: DSA showing the PICA obtained sufficient blood flow from the proximal VA, and the distal VA (arrow) occluded beyond the PICA after the FD deployment from PICA to VA. CT, computed tomography; DSA, digital subtraction angiography; EVT, endovascular treatment; FD, flow diverter; L, left; PICA, posterior inferior cerebellar artery; R, right; SAH, subarachnoid hemorrhage; VA, vertebral artery.

In VA dissections with no PICA involvement, when the contralateral VA has sufficient collateral to the BA, deconstructive EVT, including PAO and trapping of the dissection, can be used (Figure 11A) (80, 81). During reconstructive EVT, traditional stent-assisted EVT may be sufficient for small sidewall lesions (82). For large lesions, reconstructive EVT with FDs may be helpful (79). During FD deployment, adjunctive coiling may be necessary for large or fusiform ruptured dissections (Figure 11B).

In VA dissections with PICA involvement, PAO of the VA under dissection can be used. However, the PICA territory can suffer ischemia due to insufficient retrograde blood flow. Trapping dissection is the most reliable treatment, but patients who can tolerate PICA obliteration must be carefully selected. Reconstructive EVT to prevent dissection and preserve the PICA is an ideal option (83). During reconstructive EVT with traditional stenting, while preserving the PICA, dense coiling of the aneurysm is necessary. FD can decrease the necessity of coiling. In unruptured lesions, FDs can be used alone (Figure 11C).

#### 8.2.3 PCA dissection

PCA dissections are uncommon (84). They can present with ischemic symptoms in the PCA territory, mass effects, or SAH (84). For ischemic lesions, even occluded PCAs, conservative anticoagulation management can be chosen, and EVT should not be routinely recommended (85–87). For acute ruptured PCA dissections or chronic symptomatic dissections, EVT may be necessary (88). Both reconstructive and deconstructive EVT can be used. Deconstructive EVT is relatively safe even in the absence of a BOT due to rich collaterals, which especially benefit critically ill patients with ruptured PCA dissecting aneurysms or cases of difficult access or financial constraints.

**Figure 12:**
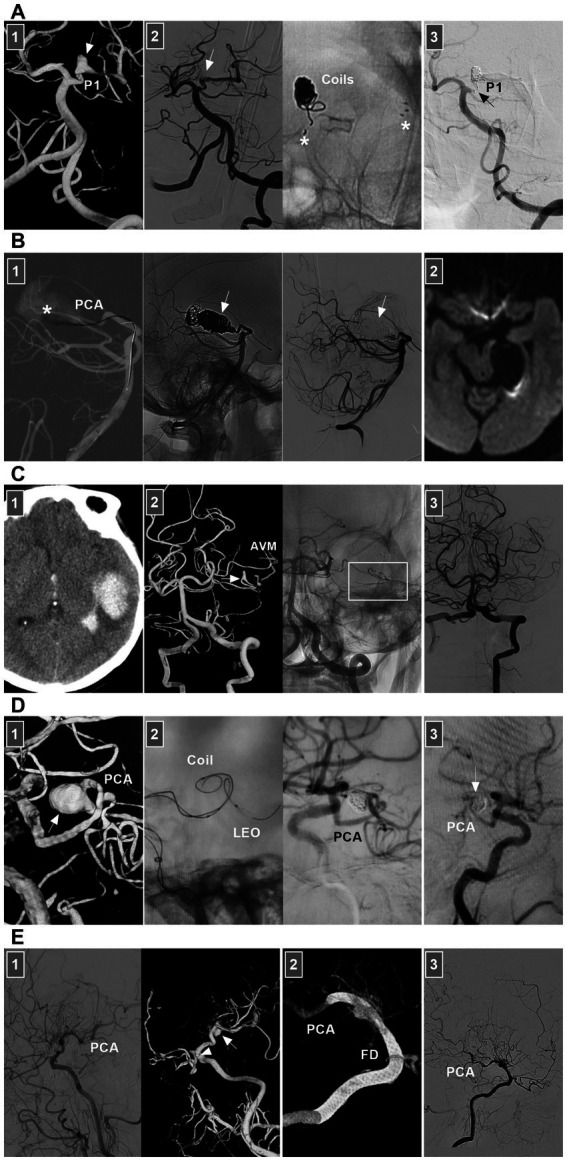
EVT of PCA dissections. **(A)** Panel 1: DSA showing a P1 dissection (arrow). Panel 2: DSA (left panel) showing that the dissection (arrow) was coiled by stent assistance, and X-ray image (right panel) showing the coils and stent (asterisks). Panel 3: Six-month follow-up DSA showing that the P1 segment of the PCA was nearly occluded (arrow). **(B)** Panel 1: X-ray image (left panel) showing the microcatheter went into the giant distal PCA dissection aneurysm (asterisk) to wait for the coiling, DSA and unsubtracted DSA (middle and right panels) images showing the aneurysm (arrows) and the parent PCA were occluded. Panel 2: Diffuse weighted image of magnetic resonance showing no ischemia of PCA region. **(C)** Panel 1: CT showing left temporal hemorrhage. Panel 2: DSA (left panel) showing an AVM supplied by the branch of PCA with a flow-related dissecting aneurysm (arrow), and unsubtracted DSA (right panel) showing the dissection was embolized by casting Onyx (frame). Panel 3: DSA showing the AVM was obliterated. **(D)** Panel 1: DSA showing a sidewall dissecting aneurysm (arrow) of distal PCA. Panel 2: X-ray image (left panel) showing the design of the EVT by LEO stent assisted-coiling, and DSA (right panel) showing the aneurysm was embolized. Panel 3: Six-month follow-up DSA showing the aneurysm (arrow) had no recurrence. **(E)** Panel 1: DSA (left panel) showing the moyamoya disease and well-developed PCA, and DSA (right panel) showing a distal PCA dissecting aneurysm and an ophthalmic aneurysm (arrowhead). Panel 2: Vaso-reconstructive image showing that the FD covered two aneurysms. Panel 3: DSA showing the PCA was patent. AVM, arteriovenous malformation; CT, computed tomography; DSA, digital subtraction angiography; EVT, endovascular treatment; FD, flow diverter; PCA, posterior cerebral artery; P1, first segment of PCA.

For P1 and P1–P2 junction dissections, because of the presence of thalamic perforating arteries, acute occlusion of the proximal PCA can be life-threatening (89). However, chronic occlusion was safe (Figure 12A). The potential collateral supply and hemodynamic balance between the anterior and posterior choroidal arteries, pericallosal vessels, and ACA and MCA to the distal PCA make P2 occlusion safe (90, 91). The P2 segment of the fetal-type PCA can send out more perforating arteries, and anastomosis between the MCA and PCA tends to result in less development of collaterals (92). At this time, PAO should be performed cautiously. Owing to the rich collateral circulation, PAO of aneurysms in the P3–P4 segment can be performed (Figure 12B) (88, 93, 94). For PCA flow-related aneurysms, reconstructive EVT is often difficult, and PAO can be performed (Figure 12C). However, PAO is associated with a nonnegligible rate of complications, even though most are minor events such as hemianopsia (95).

Compared with deconstructive EVT, reconstructive EVT is promising because current new devices have good clinical and safety profiles (91). In a report by Tang et al. (96) in 2022, braided stent-assisted coiling resulted in a high occlusion rate and a relatively low complication rate in treating PCA dissecting aneurysms. In addition to braided stents such as LVIS (Microvention, Tustin, California, United States) and LEO stents (Balt, Montmorency, France) (Figure 12D), FDs have revolutionized EVT for dissections (Figure 12E). However, FD deployment for PCA dissection is fraught with the risk of thromboembolic complications owing to side branch coverage by the FD (96).

#### 8.2.4 Superior cerebellar artery dissection

Most superior cerebellar artery (SCA) dissections do not require treatment; even rarely, ruptured dissections can resolve spontaneously (97). However, in general, for ruptured and large symptomatic SCA dissections or flow-related SCA dissections with AVMs, EVT can be suggested. The SCA is so thin that reconstructive EVT is often difficult (98, 99). Therefore, PAO must be performed in most SCA dissections. The proximal S1 segment of the SCA can send off perforators to the brainstem, and occlusion of the S1 segment can result in brainstem infarction (100). PAO should be the last resort. With the development of equipment, in recent reports, small braided stents such as LVISs and LEO stents and small-sized FDs have been used to reconstruct the SCA successfully in select cases with a thick SCA (101, 102). However, PAO for dissections of distal S2–S4 segments is safe (103).

#### 8.2.5 AICA dissection

**Figure 13:**
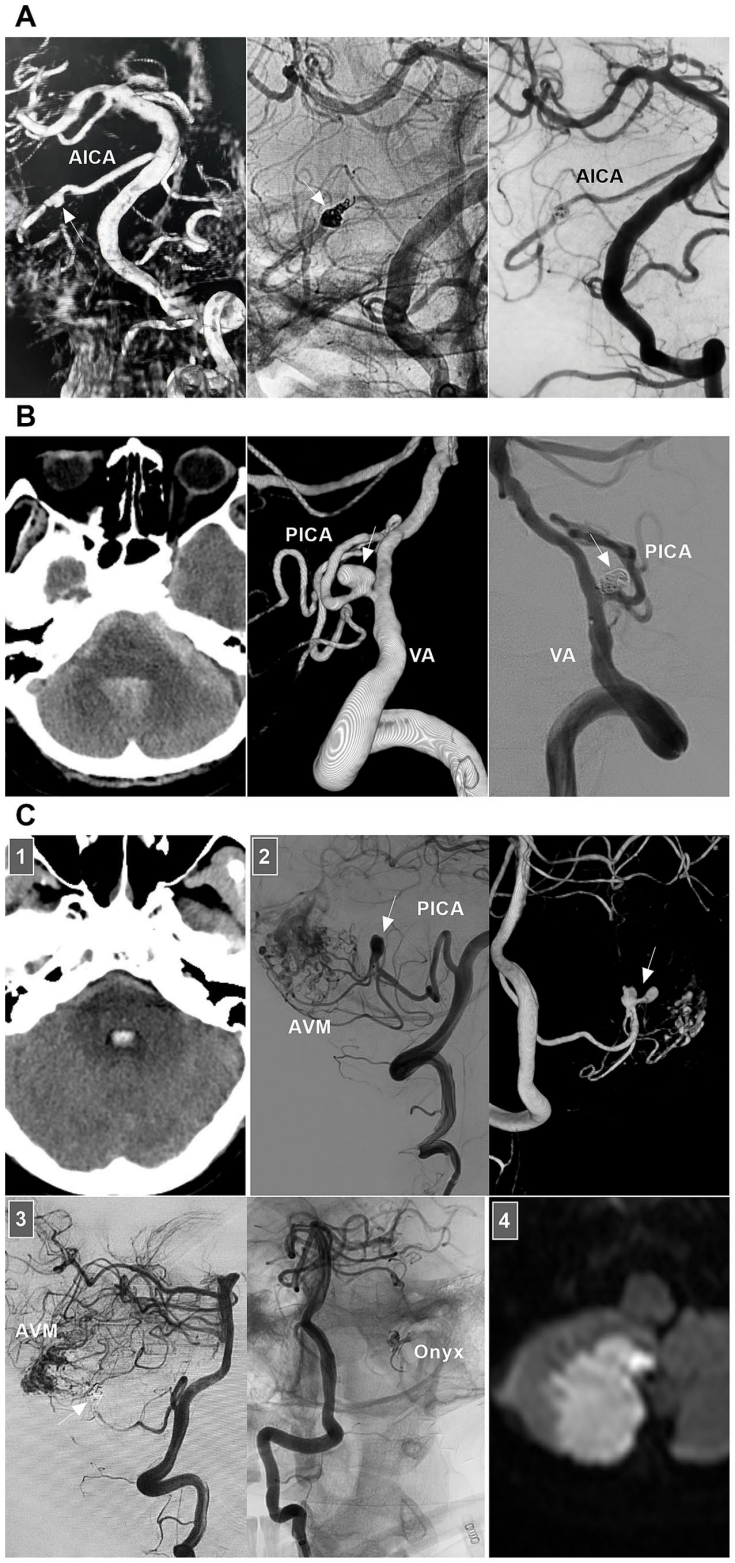
EVT of AICA and PICA dissections. **(A)** Left panel: DSA showing a ruptured AICA dissection (arrow). Middle panel: Unsubtracted DSA showing the dissection (arrow) was coiled. Right panel: DSA showing the AICA was patent, and chronic occlusion was worthy to expected. **(B)** Left panel: CT showing SAH. Middle panel: DSA showing a ruptured PCA sidewall dissecting aneurysm (arrow). Right panel: DSA showing the aneurysms (arrow) was coiled selectively. **(C)** Panel 1: CT showing the fourth ventricle hemorrhage. Panel 2: DSA (left panel) and its reconstructive image (right panel) showing an AVM that supplied by PICA with flow-related dissecting aneurysms (arrows). Panel 3: DSA (left panel) showing the aneurysms were embolized (arrow), unsubtracted DSA (right panel) showing the Onyx in the aneurysm. Panel 4: Diffuse weighted image of magnetic resonance showing asymptomatic acute ischemia of cerebellar hemisphere. AICA, anterior inferior cerebellar artery; AVM, arteriovenous malformation; CT, computed tomography; DSA, digital subtraction angiography; EVT, endovascular treatment; PICA, posterior inferior cerebellar artery; SAH, subarachnoid hemorrhage; VA, vertebral artery.

For ruptured AICA dissections with a risk of rebleeding or flow-related dissection with AVMs, EVT may be suggested. The AICA is a small artery that can be divided into the a1–a4 segments (104, 105). The proximal a1 segment sends off brainstem perforators, and the a2 segment sends off the internal auditory artery. Proximal PAO can result in brainstem infarction and hearing loss, and it is the last resort (104, 106). Reconstructive EVT was the preferred choice. Owing to the limitation of the AICA diameter, stenting in the AICA can be employed only for a thick AICA or the common trunk of the AICA-PICA (107). For example, in 2024, Kass-Hout et al. (108) treated AICA dissection with FD [a silk vista baby device (Balt, Montmorency, France)]. PAO is acceptable for distal AICA dissections (Figure 13A).

#### 8.2.6 PICA

For unruptured PICA dissections, conservative treatment can be recommended for those without obvious angiographic risk factors for hemorrhage, such as pseudoaneurysm (109). However, for ruptured PICA dissections with a high risk of rebleeding, recurrence, or flow-related AVM, EVT may be suggested (Figures 13B,C). Ideal EVT comprises complete embolization of the PICA dissections while preserving the PICA and perforating arteries (110–112). However, reconstructive EVT is difficult to perform because the diameter of the PICA trunk is not thick. Therefore, PAO had to be employed.

For proximal P1 aneurysms, PAO is dangerous because of brainstem infarction (113–115). However, Malcolm et al. (116) reported in 2020 that the risk of brainstem stroke from proximal PICA sacrifice may not be as high as expected. PAO for proximal PICA dissection can be used only as a last resort for poor surgical candidates or those with good collateral perfusion. However, owing to the good possibility of collateral flow, PAO for distal P2–P5 dissections can be safely performed (117, 118). For PICAs with a double origin or with PICA communicating arteries, aggressive PAO can be performed (118, 119).

Recently, with the advancement of small low-profile stents, a proximal PICA trunk that is not too thin can be reconstructed by stenting. In 2022, Lim et al. (109) reported that a ruptured proximal PICA dissection was reconstructed via an LIVS junior stent, and follow-up angiography confirmed that the dissection was cured. In addition, small very low-profile FDs, such as the silk Vista Baby device, are suitable for use with a 0.017-inch microcatheter and can be deployed without the need for more support; thus, they can be used to treat proximal PICA dissection (120). In addition, unruptured dissection of the proximal PICA, covering the PICA orifice with an FD, or even the use of a low-metal-coverage stent, can result in dissection regression by reducing arterial flow (121, 122).

## 9 Conclusion

It was feasible to consider that the following IADs were stable: unruptured, small, fusiform, and not flow related. Ruptured IADs and unruptured IADs with acute large artery occlusion and aneurysms that increased in size or caused compression and therefore a mass effect during the follow-up period required intervention. EVT is currently the treatment of choice for IADs. Because IADs are relatively complex diseases, a personalized choice of deconstructive or reconstructive EVT is necessary depending on the type of dissection, symptoms, and location. Then, good outcomes can be obtained.

## References

[1] Debette S Compter A Labeyrie MA Uyttenboogaart M Metso TM Majersik JJ. Epidemiology, pathophysiology, diagnosis, and management of intracranial artery dissection. Lancet Neurol. (2015) 14:640–54. doi: 10.1016/S1474-4422(15)00009-5, PMID: 25987283

[2] Prestes MZ Oliveira LB Soares C Ramos de Souza J Geris da Costa J Rabelo NN. Sole stenting versus stent-assisted coiling for treating dissecting posterior circulation aneurysms: a systematic review and meta-analysis. World Neurosurg. (2024) 192:201–211.e9. doi: 10.1016/j.wneu.2024.09.020, PMID: 39270787

[3] So TY Mitchell PJ Dowling RJ Yan B. Efficacy, complications and clinical outcome of endovascular treatment for intracranial intradural arterial dissections. Clin Neurol Neurosurg. (2014) 117:6–11. doi: 10.1016/j.clineuro.2013.11.015, PMID: 24438797

[4] Yamaura A Yoshimoto T Hashimoto N Ono JI. Nationwide study of nontraumatic intracranial arterial dissection: treatment and its results. Surg Cereb Stroke. (1998) 26:87–95. doi: 10.2335/scs1987.26.2_87

[5] Page MJ McKenzie JE Bossuyt PM Boutron I Hoffmann TC Mulrow CD. The PRISMA 2020 statement: an updated guideline for reporting systematic reviews. BMJ. (2021) 372:n71. doi: 10.1136/bmj.n71, PMID: 33782057 PMC8005924

[6] Pelkonen O Tikkakoski T Leinonen S Pyhtinen J Sotaniemi K. Intracranial arterial dissection. Neuroradiology. (1998) 40:442–7. doi: 10.1007/s002340050620 9730344

[7] Debette S Mazighi M Bijlenga P Pezzini A Koga M Bersano A. ESO guideline for the management of extracranial and intracranial artery dissection. Eur Stroke J. (2021) 6:XXXIX–LXXXVIII. doi: 10.1177/23969873211046475, PMID: 34746432 PMC8564160

[8] Mizutani T. A fatal, chronically growing basilar artery: a new type of dissecting aneurysm. J Neurosurg. (1996) 84:962–71. doi: 10.3171/jns.1996.84.6.0962, PMID: 8847591

[9] Mizutani T. Natural course of intracranial arterial dissections. J Neurosurg. (2011) 114:1037–44. doi: 10.3171/2010.9.JNS10668, PMID: 20950090

[10] Rajpal G Naik V. Management of intracranial arterial dissection. Neurol India. (2018) 66:40–2. doi: 10.4103/0028-3886.222834, PMID: 29322954

[11] Huh CW Jin SC. Ruptured dissecting aneurysm in communicating internal carotid artery segments treated using a stent-assisted endovascular technique. Interv Neuroradiol. (2018) 24:130–4. doi: 10.1177/1591019917747244, PMID: 29357768 PMC5847009

[12] Rodríguez-Hernández A Zador Z Rodríguez-Mena R Lawton MT. Distal aneurysms of intracranial arteries: application of numerical nomenclature, predilection for cerebellar arteries, and results of surgical management. World Neurosurg. (2013) 80:103–12. doi: 10.1016/j.wneu.2012.09.010, PMID: 23017587

[13] Fukuda N Kanemaru K Hashimoto K Yoshioka H Senbokuya N Yagi T. Embolization of a peripheral cerebral aneurysm associated with intracranial major artery occlusion through a transdural anastomotic artery: case report. Interv Neuroradiol. (2019) 25:172–6. doi: 10.1177/1591019918801539, PMID: 30231796 PMC6448377

[14] Wiedmann MKH Davidoff C Lo Presti A Ni W Rhim JK Simons M. Treatment of ruptured aneurysms of the choroidal collateral system in moyamoya disease: a systematic review and data analysis. J Neurosurg. (2022) 136:637–46. doi: 10.3171/2021.1.JNS203936, PMID: 34450582

[15] Yoshimoto Y Wakai S. Unruptured intracranial vertebral artery dissection. Clinical course and serial radiographic imagings. Stroke. (1997) 28:370–4. doi: 10.1161/01.STR.28.2.370, PMID: 9040692

[16] Li S Yan B Kaye A Mitchell P Dowling R Collins M. Prognosis of intracranial dissection relates to site and presenting features. J Clin Neurosci. (2011) 18:789–93. doi: 10.1016/j.jocn.2010.11.006, PMID: 21507658

[17] Hosogai M Matsushige T Shimonaga K Kawasumi T Kurisu K Sakamoto S. Stent-assisted coil embolization for ruptured intracranial dissecting aneurysms involving essential vessels. World Neurosurg. (2018) 119:e728–33. doi: 10.1016/j.wneu.2018.07.254, PMID: 30098438

[18] Urasyanandana K Songsang D Aurboonyawat T Chankaew E Withayasuk P Churojana A. Treatment outcomes in cerebral artery dissection and literature review. Interv Neuroradiol. (2018) 24:254–62. doi: 10.1177/1591019918755692, PMID: 29433365 PMC5967189

[19] Rahme RJ Aoun SG McClendon J Jr El Ahmadieh TY Bendok BR. Spontaneous cervical and cerebral arterial dissections: diagnosis and management. Neuroimaging Clin N Am. (2013) 23:661–71. doi: 10.1016/j.nic.2013.03.013 24156857

[20] Mizutani T. Middle cerebral artery dissecting aneurysm with persistent patent pseudolumen. Case report. J Neurosurg. (1996) 84:267–8. doi: 10.3171/jns.1996.84.2.0267, PMID: 8592231

[21] Niikawa S Yamada J Sumi Y Yamakawa H. Dissecting aneurysm of the middle cerebral artery manifesting as subarachnoid hemorrhage and hemorrhagic infarctions. Case report. Neurol Med Chir. (2002) 42:62–6. doi: 10.2176/nmc.42.62 11944591

[22] Krings T Choi IS. The many faces of intracranial arterial dissections. Interv Neuroradiol. (2010) 16:151–60. doi: 10.1177/159101991001600206, PMID: 20642889 PMC3277975

[23] Lin CY Chen HC Wu YH. Using high-resolution vessel wall magnetic resonance images in a patient of intracranial artery dissection related acute infarction. Diagnostics. (2024) 14:1463. doi: 10.3390/diagnostics14141463, PMID: 39061600 PMC11276065

[24] Enoki T Kida K Jomoto W Kawanaka Y Shirakawa M Miyama M. 3D phase-sensitive inversion recovery sequence for intracranial vertebrobasilar artery dissection. J Clin Neurosci. (2023) 118:52–7. doi: 10.1016/j.jocn.2023.10.008, PMID: 37871475

[25] Bae DW Lee JH Shin JH Ihn YK Sung JH. Detection of cerebral aneurysm and intracranial vertebral dissection using non-enhanced magnetic resonance imaging in emergency setting: emphasis on magnitude image of susceptibility-weighted image. Interv Neuroradiol. (2023) 29:665–73. doi: 10.1177/15910199221104613, PMID: 35642276 PMC10680967

[26] Tang M Gao J Gao J Yan X Zhang X Li L. Evaluating intracranial artery dissection by using three-dimensional simultaneous non-contrast angiography and intra-plaque hemorrhage high-resolution magnetic resonance imaging: a retrospective study. Acta Radiol. (2022) 63:401–9. doi: 10.1177/0284185121992235, PMID: 33601894

[27] Hwang JW Jung JM Cha JH Jung IE Park MH Kwon DY. Using the region of interest from time-of-flight magnetic resonance angiography to differentiate between intracranial arterial dissection and true atherosclerotic stenosis. Cerebrovasc Dis. (2019) 47:8–14. doi: 10.1159/000496505, PMID: 30726839

[28] Kim JW Shin NY Kim YD Lee SK Lim SM Oh SW. Added value of 3D proton-density weighted images in diagnosis of intracranial arterial dissection. PLoS One. (2016) 11:e0166929. doi: 10.1371/journal.pone.0166929, PMID: 27880798 PMC5120794

[29] Demerath T Bonati L El Mekabaty A Schubert T. High-resolution compressed-sensing time-of-flight MRA in a case of acute ICA/MCA dissection. Neuroradiology. (2020) 62:753–6. doi: 10.1007/s00234-020-02395-y, PMID: 32198564

[30] Matsukawa S Ishii A Fushimi Y Terada Y Natsuhara H Kikuchi T. Ruptured thrombosed vertebral artery dissecting aneurysm treated with staged flow diverter after prediction of the rupture point by vessel wall MRI. Neuroradiol J. (2024) 37:772–5. doi: 10.1177/19714009231224420, PMID: 38148669 PMC11531014

[31] Zhang Y Sui B Liu J Wang Y Tian Z Chen J. Aneurysm wall enhancement on magnetic resonance imaging as a risk factor for progression of unruptured vertebrobasilar dissecting aneurysms after reconstructive endovascular treatment. J Neurosurg. (2018) 128:747–55. doi: 10.3171/2016.11.JNS162433, PMID: 28387631

[32] Leira EC. Dedicated guidelines for arterial dissections: more specifics amid uncertainty. Stroke. (2022) 53:e53–5. doi: 10.1161/STROKEAHA.121.037324, PMID: 34937422

[33] Egashira S Kunisawa S Koga M Ihara M Tsuruta W Uesaka Y. Safety and outcomes of intravenous thrombolysis in acute ischemic stroke with intracranial artery dissection. Int J Stroke. (2025). doi: 10.1177/17474930251317326, PMID: 39834052

[34] Shimizu H Ono T Abe T Hokari M Egashira Y Shimonaga K. Current treatment results of intracranial carotid artery dissection causing cerebral ischemia: a Japanese Nationwide survey. Neurol Med Chir. (2023) 63:80–9. doi: 10.2176/jns-nmc.2022-0249, PMID: 36599430 PMC9995147

[35] Al-Mufti F Kamal N Damodara N Nuoman R Gupta R Alotaibi NM. Updates in the management of cerebral infarctions and subarachnoid hemorrhage secondary to intracranial arterial dissection: a systematic review. World Neurosurg. (2019) 121:51–8. doi: 10.1016/j.wneu.2018.09.153, PMID: 30268550

[36] Tsukada T Izumi T Isoda H Nishihori M Kropp AE Mizuno T. Comparison of hemodynamic stress in healthy vessels after parent artery occlusion and flow diverter stent treatment for internal carotid artery aneurysm. J Neurosurg. (2022) 136:619–26. doi: 10.3171/2021.2.JNS204185, PMID: 34416714

[37] Brunozzi D See A Rizko M Choi J Atwal G Alaraj A. Impact of cerebral aneurysm size on distal intracranial hemodynamics and changes following flow diversion. Interv Neuroradiol. (2022) 28:291–5. doi: 10.1177/15910199211032467, PMID: 34425691 PMC9185094

[38] Kim DJ Kim BM Suh SH Kim DI. Self-expanding stent placement for anterior circulation intracranial artery dissection presenting with ischemic symptoms. Neurosurgery. (2015) 76:158–64. doi: 10.1227/NEU.0000000000000582, PMID: 25549188

[39] Labeyrie MA Civelli V Reiner P Aymard A Saint-Maurice JP Zetchi A. Prevalence and treatment of spontaneous intracranial artery dissections in patients with acute stroke due to intracranial large vessel occlusion. J Neurointerv Surg. (2018) 10:761–4. doi: 10.1136/neurintsurg-2018-013763, PMID: 29511116

[40] Cho WC Lee HJ Choi JH Lee KS Kim BS Shin YS. Clinical and radiological outcomes of vertebral artery dissecting aneurysms treated with endovascular treatments: a 12-year single-center experience. World Neurosurg. (2023) 175:e904–13. doi: 10.1016/j.wneu.2023.04.040, PMID: 37075898

[41] Amoukhteh M Hassankhani A Valizadeh P Jannatdoust P Ghozy S Kobeissi H. Flow diverters in the treatment of intracranial dissecting aneurysms: a systematic review and meta-analysis of safety and efficacy. J Neurointerv Surg. (2024) 16:1005–12. doi: 10.1136/jnis-2023-021117, PMID: 38212103

[42] Monteiro A Khan A Donnelly BM Kuo CC Burke SM Waqas M. Treatment of ruptured intracranial aneurysms using the novel generation of flow-diverters with surface modification: a systematic review and meta-analysis. Interv Neuroradiol. (2024) 30:350–60. doi: 10.1177/15910199221117921, PMID: 35929825 PMC11310723

[43] Essibayi MA Lanzino G Keser Z. Endovascular treatments of intracranial vertebral and internal carotid arteries dissections: an interactive systematic review and meta-analysis. Interv Neuroradiol. (2024) 30:22–30. doi: 10.1177/15910199221095789, PMID: 35450460 PMC10956451

[44] Amoukhteh M Hassankhani A Jannatdoust P Valizadeh P Ghozy S Bilgin C. Comparative meta-analysis of endovascular strategies for intracranial dissecting aneurysms: flow diverters versus stents with or without coiling. Interv Neuroradiol. (2024):15910199241262070. doi: 10.1177/15910199241262070, PMID: 38873695 PMC11571147

[45] Brenner LO Prestes MZ Soares C Romeiro P Gomez VA Rabelo NN. Flow diverter versus stent-assisted coiling treatment for managing dissecting intracranial aneurysms: a systematic review and meta-analysis. Interv Neuroradiol. (2024). doi: 10.1177/15910199241301820, PMID: 39668743 PMC11638934

[46] Chaves C Estol C Esnaola MM Gorson K O’Donoghue M De Witt LD. Spontaneous intracranial internal carotid artery dissection: report of 10 patients. Arch Neurol. (2002) 59:977–81. doi: 10.1001/archneur.59.6.977, PMID: 12056934

[47] Kim J Chang C Jung Y. Selective coil embolization of ruptured fusiform aneurysm involving anterior choroidal artery and posterior communicating artery. World Neurosurg. (2018) 118:274–8. doi: 10.1016/j.wneu.2018.07.135, PMID: 30053567

[48] Surdell DL Bernstein RA Hage ZA Batjer HH Bendok BR. Symptomatic spontaneous intracranial carotid artery dissection treated with a self-expanding intracranial nitinol stent: a case report. Surg Neurol. (2009) 71:604–9. doi: 10.1016/j.surneu.2007.11.021, PMID: 18313734

[49] Jeon P Kim BM Kim DI Shin YS Kim KH Park SI. Emergent self-expanding stent placement for acute intracranial or extracranial internal carotid artery dissection with significant hemodynamic insufficiency. AJNR Am J Neuroradiol. (2010) 31:1529–32. doi: 10.3174/ajnr.A2115, PMID: 20430849 PMC7966117

[50] Ogiwara H Maeda K Hara T Kimura T Abe H. Spontaneous intracranial internal carotid artery dissection treated by intra-arterial thrombolysis and superficial temporal artery-middle cerebral artery anastomosis in the acute stage-case report. Neurol Med Chir. (2005) 45:148–51. doi: 10.2176/nmc.45.148, PMID: 15782006

[51] Kadooka K Tanaka M Shimada K Hadeishi H. Subarachnoid hemorrhage from dissecting aneurysm of the posterior communicating artery. J Clin Neurosci. (2016) 32:125–8. doi: 10.1016/j.jocn.2015.12.040, PMID: 27343043

[52] Nounaka Y Murai Y Shirokane K Matano F Koketsu K Nakae R. Spontaneous middle cerebral artery dissection: a series of six cases and literature review. Neurosurg Rev. (2023) 46:229. doi: 10.1007/s10143-023-02139-5, PMID: 37676338

[53] De Jesus O Lugo Morales F Vicenty JC. Pseudoaneurysm formation in a pediatric patient after non-traumatic middle cerebral artery dissection with a rapid spontaneous complete thrombosis. Cureus. (2022) 14:e32251. doi: 10.7759/cureus.32251, PMID: 36620827 PMC9814825

[54] Furst T Ellens NR Bender MT Mattingly TK. Ischemic stroke caused by spontaneous anterior circulation intracranial arterial dissections: patient series. J Neurosurg Case Lessons. (2023) 5:CASE22564. doi: 10.3171/CASE22564, PMID: 36880515 PMC10550662

[55] Nam DH Park SK. Endovascular treatment in ruptured middle cerebral artery dissection preservation of arterial continuity. J Cerebrovasc Endovasc Neurosurg. (2015) 17:108–12. doi: 10.7461/jcen.2015.17.2.108, PMID: 26157690 PMC4495084

[56] Zhao P Zhu D Wen W Zhou Y Fang Y Li Q. Endovascular treatment of middle cerebral artery dissecting aneurysms: a 7-year single-center study. World Neurosurg. (2018) 112:e119–24. doi: 10.1016/j.wneu.2017.12.153, PMID: 29355801

[57] Baltacioğlu F Cekirge S Saatci I Oztürk H Arat A Pamir N. Distal middle cerebral artery aneurysms. Endovascular treatment results with literature review. Interv Neuroradiol. (2002) 8:399–407. doi: 10.1177/159101990200800409, PMID: 20594501 PMC3572496

[58] Kivipelto L Niemelä M Meling T Lehecka M Lehto H Hernesniemi J. Bypass surgery for complex middle cerebral artery aneurysms: impact of the exact location in the MCA tree. J Neurosurg. (2014) 120:398–408. doi: 10.3171/2013.10.JNS13738, PMID: 24286147

[59] Nagamine Y Fukuoka T Hayashi T Kato Y Deguchi I Maruyama H. Research article: clinical characteristics of isolated anterior cerebral artery territory infarction due to arterial dissection. J Stroke Cerebrovasc Dis. (2014) 23:2907–13. doi: 10.1016/j.jstrokecerebrovasdis.2014.07.017, PMID: 25280818

[60] Ohkuma H Suzuki S Kikkawa T Shimamura N. Neuroradiologic and clinical features of arterial dissection of the anterior cerebral artery. AJNR Am J Neuroradiol. (2003) 24:691–9. PMID: 12695205 PMC8148685

[61] Thines L Zairi F Taschner C Leclerc X Lucas C Bourgeois P. Subarachnoid hemorrhage from spontaneous dissection of the anterior cerebral artery. Cerebrovasc Dis. (2006) 22:452–6. doi: 10.1159/000095383, PMID: 16940718

[62] Asano S Hara T. Chronic recanalization of dissection of the distal anterior cerebral artery: case report and review of the literature. Case Rep Med. (2009) 2009:303695. doi: 10.1155/2009/303695, PMID: 19724653 PMC2734918

[63] Iwasaki M Hattori I Sasaki M Ishimori H Nemoto A Hikita C. Stent-assisted coil embolization for anterior cerebral artery dissection presented with cerebral infarction. Surg Neurol Int. (2015) 6:182. doi: 10.4103/2152-7806.171240, PMID: 26677416 PMC4681129

[64] Lv X Li Y Jiang C Jiang P Wu Z. Dissecting aneurysm at the proximal anterior cerebral artery treated by parent artery occlusion. Interv Neuroradiol. (2009) 15:123–6. doi: 10.1177/159101990901500121, PMID: 20465942 PMC3306144

[65] Giorgianni A Pellegrino C Minotto R Mercuri A Frattini L Baruzzi F. Flow-diverter stenting of post-traumatic bilateral anterior cerebral artery pseudoaneurysm: a case report. Interv Neuroradiol. (2015) 21:23–8. doi: 10.1177/1591019915575441, PMID: 25934771 PMC4757201

[66] Conte M Cagil E Lanzino G Keser Z. Fusiform aneurysms of anterior cerebral artery: center experience and systematic literature review. Neurosurg Rev. (2023) 47:11. doi: 10.1007/s10143-023-02247-2, PMID: 38087068

[67] Moon HS Kim TS Joo SP. Surgical treatment of giant serpentine aneurysm of A2–A3 segment distal anterior cerebral artery: technical case report. J Korean Neurosurg Soc. (2012) 52:501–4. doi: 10.3340/jkns.2012.52.5.501, PMID: 23323176 PMC3539090

[68] Alurkar A Karanam LS Oak S Nayak S. Endovascular treatment of fusiform A2 aneurysm with parent artery occlusion. Surg Neurol Int. (2014) 5:199–202. doi: 10.4103/2152-7806.137752, PMID: 25184100 PMC4138823

[69] Kim ST Jeong YG Jeong HW. Treatment of a Giant serpentine aneurysm in the anterior cerebral artery. J Cerebrovasc Endovasc Neurosurg. (2016) 18:141–6. doi: 10.7461/jcen.2016.18.2.141, PMID: 27790407 PMC5081501

[70] Hou K Wang Y Li W Yu J. Endovascular treatment of brain arteriovenous malformations involving the anterior cerebral artery. Med Int. (2021) 1:22. doi: 10.3892/mi.2021.22, PMID: 36698539 PMC9829091

[71] Yoshimoto Y Hoya K Tanaka Y Uchida T. Basilar artery dissection. J Neurosurg. (2005) 102:476–81. doi: 10.3171/jns.2005.102.3.0476, PMID: 15796382

[72] Nakatomi H Nagata K Kawamoto S Furusho JI. Basilar artery occlusion due to spontaneous basilar artery dissection in a child. Acta Neurochir. (1999) 141:99–104. doi: 10.1007/s007010050272, PMID: 10071693

[73] Borota L Libard S Fahlström M Latini F Lundström E. Complete functional recovery in a child after endovascular treatment of basilar artery occlusion caused by spontaneous dissection: a case report. Childs Nerv Syst. (2022) 38:1605–12. doi: 10.1007/s00381-021-05428-w, PMID: 34893933 PMC9325841

[74] Komiyama M Yoshimura M Honnda Y Matsusaka Y Yasui T. Acute basilar artery dissection treated by emergency stenting in a 13-year-old boy. Pediatr Neurosurg. (2005) 41:318–22. doi: 10.1159/000088734 16293951

[75] Yu J. Current research status and future of endovascular treatment for basilar artery aneurysms. Neuroradiol J. (2024) 37:571–86. doi: 10.1177/19714009241242584, PMID: 38560789 PMC11528780

[76] Kant S Goel V Garg A Sebastian LJD. Giant dissecting aneurysm of basilar artery in a child—treated by flow reversal: a case report. Interv Neuroradiol. (2023). doi: 10.1177/15910199231154688, PMID: 36734092 PMC12475313

[77] Mizutani T Aruga T Kirino T Miki Y Saito I Tsuchida T. Recurrent subarachnoid hemorrhage from untreated ruptured vertebrobasilar dissecting aneurysms. Neurosurgery. (1995) 36:905–13. doi: 10.1227/00006123-199505000-00003, PMID: 7791980

[78] Takagi T Takayasu M Suzuki Y Yoshida J. Prediction of rebleeding from angiographic features in vertebral artery dissecting aneurysms. Neurosurg Rev. (2007) 30:32–9. doi: 10.1007/s10143-006-0049-1, PMID: 17061136

[79] Lee W Han HJ Kim J Park KY Kim YB Jang CK. Flow diverter for the treatment of large (>10 mm) vertebral artery dissecting aneurysms. Acta Neurochir. (2022) 164:1247–54. doi: 10.1007/s00701-021-04965-2, PMID: 34383115

[80] Lehto H Niemelä M Kivisaari R Laakso A Jahromi BR Hijazy F. Intracranial vertebral artery aneurysms: clinical features and outcome of 190 patients. World Neurosurg. (2015) 84:380–9. doi: 10.1016/j.wneu.2015.03.034, PMID: 25828051

[81] Kim BM Shin YS Kim SH Suh SH Ihn YK Kim DI. Incidence and risk factors of recurrence after endovascular treatment of intracranial vertebrobasilar dissecting aneurysms. Stroke. (2011) 42:2425–30. doi: 10.1161/STROKEAHA.111.617381, PMID: 21778439

[82] Kyeung Ko J Weon Lee S Hwa Choi C Lee TH. Endovascular reconstructive treatment using a fill-and-tunnel technique for a fusiform vertebral artery dissecting aneurysm with ipsilateral dominance. Interv Neuroradiol. (2019) 25:539–47. doi: 10.1177/1591019919846616, PMID: 31088243 PMC6777117

[83] Chen JA Garrett MC Mlikotic A Ausman JI. Treatment of intracranial vertebral artery dissecting aneurysms involving the posterior inferior cerebellar artery origin. Surg Neurol Int. (2019) 10:116. doi: 10.25259/SNI-281-2019, PMID: 31528452 PMC6744774

[84] Nistri M Perrini P Di Lorenzo N Cellerini M Villari N Mascalchi M. Third-nerve palsy heralding dissecting aneurysm of posterior cerebral artery: digital subtraction angiography and magnetic resonance appearance. J Neurol Neurosurg Psychiatry. (2007) 78:197–8. doi: 10.1136/jnnp.2006.098129, PMID: 17229750 PMC2077667

[85] Sabben C Charbonneau F Delvoye F Strambo D Heldner MR Ong E. Endovascular therapy or medical management alone for isolated posterior cerebral artery occlusion: a multicenter study. Stroke. (2023) 54:928–37. doi: 10.1161/STROKEAHA.122.042283, PMID: 36729389

[86] Räty S Nguyen TN Nagel S Strambo D Michel P Herweh C. Endovascular thrombectomy versus intravenous thrombolysis of posterior cerebral artery occlusion stroke. J Stroke. (2024) 26:290–9. doi: 10.5853/jos.2024.00458, PMID: 38836276 PMC11164587

[87] Nguyen TN Qureshi MM Strambo D Strbian D Räty S Herweh C. Endovascular versus medical management of posterior cerebral artery occlusion stroke: the PLATO study. Stroke. (2023) 54:1708–17. doi: 10.1161/STROKEAHA.123.042674, PMID: 37222709

[88] Hou K Lv X Yu J. Endovascular treatment of posterior cerebral artery trunk aneurysm: the status quo and dilemma. Front Neurol. (2021) 12:746525. doi: 10.3389/fneur.2021.746525, PMID: 35069405 PMC8778581

[89] Yoshida J Akamatsu Y Kojima D Miyoshi K Kashimura H Ogasawara K. Endovascular intervention for bilateral paramedian thalamic stroke due to occlusion of the unilateral P1 segment of the posterior cerebral artery: illustrative cases. J Neurosurg Case Lessons. (2022) 4:CASE22152. doi: 10.3171/CASE22152, PMID: 35855009 PMC9274292

[90] Hallacq P Piotin M Moret J. Endovascular occlusion of the posterior cerebral artery for the treatment of p2 segment aneurysms: retrospective review of a 10-year series. AJNR Am J Neuroradiol. (2002) 23:1128–36. PMID: 12169469 PMC8185732

[91] Essibayi MA Oushy SH Keser Z Lanzino G. Natural history and management of posterior cerebral artery aneurysms: a systematic review and meta-analysis of individual patient data. Neurosurg Rev. (2022) 45:3595–608. doi: 10.1007/s10143-022-01867-4, PMID: 36222943

[92] Xu J Xu L Wu Z Chen X Yu J Zhang J. Fetal-type posterior cerebral artery: the pitfall of parent artery occlusion for ruptured P₂ segment and distal aneurysms. J Neurosurg. (2015) 123:906–14. doi: 10.3171/2014.9.JNS1442, PMID: 25768832

[93] Luo Q Wang H Xu K Yu J. Endovascular treatments for distal posterior cerebral artery aneurysms. Turk Neurosurg. (2012) 22:141–7. doi: 10.5137/1019-5149.JTN.4079-11.0, PMID: 22437286

[94] Arat A Islak C Saatci I Kocer N Cekirge S. Endovascular parent artery occlusion in large-giant or fusiform distal posterior cerebral artery aneurysms. Neuroradiology. (2002) 44:700–5. doi: 10.1007/s00234-002-0747-5, PMID: 12185549

[95] Sturiale CL De Waure C Della Pepa GM Calabrò GE Albanese A D’Argento F. Endovascular treatment of the posterior cerebral artery aneurysms: single-center experience and a systematic review. World Neurosurg. (2016) 91:154–62. doi: 10.1016/j.wneu.2016.03.083, PMID: 27062918

[96] Tang H Shang C Zhang G Zuo Q Zhang X Xu F. Braided stents assisted coiling for endovascular management of posterior cerebral artery aneurysms: a preliminary mid-term experience. Neuroradiology. (2022) 64:1847–56. doi: 10.1007/s00234-022-02956-3, PMID: 35441874

[97] Alami B Boujraf S Maaroufi M Alaoui-Lamrani MY. Spontaneous resolution of ruptured dissecting superior cerebellar artery aneurysm. Neurol Sci. (2021) 42:1593–5. doi: 10.1007/s10072-020-04835-2, PMID: 33089475

[98] Acik V Daglioglu E Akmangit I Alagoz F Sayin B Arat A. Endovascular treatment of superior cerebellar artery aneurysms. Turk Neurosurg. (2019) 29:564–9. doi: 10.5137/1019-5149.JTN.24640-18.3, PMID: 30829386

[99] Velioglu M Selcuk H Kizilkilic O Basekim C Kocer N Islak C. Endovascular management of superior cerebellar artery aneurysms: mid and long-term results. Turk Neurosurg. (2015) 25:526–31. doi: 10.5137/1019-5149.JTN.8611-13.0, PMID: 26242327

[100] Kim CH Cho YD Jung SC Ahn JH Kang HS Kim JE. Endovascular treatment for superior cerebellar artery aneurysms: morphological features, technique, and outcome. Neuroradiology. (2014) 56:647–54. doi: 10.1007/s00234-014-1375-6, PMID: 24810727

[101] Onishi S Sakamoto S Sadatomo T Hara T Ochiai J Yuki K. Endovascular coil embolization with low-profile visualized intraluminal support junior stent for ruptured dissecting aneurysm of proximal superior cerebellar artery-case report and literature review. World Neurosurg. (2019) 122:102–5. doi: 10.1016/j.wneu.2018.10.137, PMID: 30391607

[102] Anil G Sein L Nga V Teo K Chou N Yeo TT. Dissecting distal cerebellar artery aneurysms: options beyond a parent vessel sacrifice. Neurosurg Rev. (2020) 43:771–80. doi: 10.1007/s10143-019-01119-y, PMID: 31144196

[103] Hou K Li G Wang X Xu K Yu J. Endovascular treatment for peripheral superior cerebellar artery aneurysms: current state and future considerations. World Neurosurg. (2019) 127:423–33. doi: 10.1016/j.wneu.2019.04.145, PMID: 31028980

[104] Hou K Li G Xu B Xu K Yu J. Which patients with aneurysms involving the a1–a2 segment of the anterior inferior cerebellar artery would benefit from parent artery occlusion? World Neurosurg. (2019) 126:301–9. doi: 10.1016/j.wneu.2019.03.070, PMID: 30885868

[105] Hou K Li G Luan T Xu K Xu B Yu J. Anatomical study of anterior inferior cerebellar artery and its reciprocal relationship with posterior inferior cerebellar artery based on angiographic data. World Neurosurg. (2020) 133:e459–72. doi: 10.1016/j.wneu.2019.09.047, PMID: 31526888

[106] Hou K Xu K Yu J. Endovascular treatment of anterior inferior cerebellar artery trunk aneurysms. Interv Neuroradiol. (2022) 28:604–12. doi: 10.1177/15910199211049054, PMID: 34775860 PMC9511622

[107] Ku JC Chavda V Palmisciano P Pasarikovski CR Yang VXD Kiwan R. Endovascular treatment for anterior inferior cerebellar artery-posterior inferior cerebellar artery (AICA-PICA) common trunk variant aneurysms: technical note and literature review. J Cerebrovasc Endovasc Neurosurg. (2023) 25:452–61. doi: 10.7461/jcen.2023.E2022.10.011, PMID: 37041684 PMC10774679

[108] Kass-Hout O Darkhabani Z Becske T. A rare dissecting anterior inferior cerebellar artery aneurysm treated with flow diversion using a silk vista baby device. Interv Neuroradiol. (2024). doi: 10.1177/15910199241227467, PMID: 38264953 PMC11577331

[109] Lim S Lee K Park H Heo W Hwang SH. Overlapping pure LIVS Jr. stents for isolated ruptured dissecting aneurysm of the proximal posterior inferior cerebellar artery. Medicina. (2022) 58:240. doi: 10.3390/medicina58020240 35208564 PMC8878417

[110] Horiuchi T Tanaka Y Hongo K Nitta J Kusano Y Kobayashi S. Characteristics of distal posteroinferior cerebellar artery aneurysms. Neurosurgery. (2003) 53:589–96. doi: 10.1227/01.NEU.0000079493.50657.1D, PMID: 12943575

[111] Chalouhi N Jabbour P Starke RM Tjoumakaris SI Gonzalez LF Witte S. Endovascular treatment of proximal and distal posterior inferior cerebellar artery aneurysms. J Neurosurg. (2013) 118:991–9. doi: 10.3171/2012.12.JNS121240, PMID: 23350778

[112] Lehto H Harati A Niemelä M Dashti R Laakso A Elsharkawy A. Distal posterior inferior cerebellar artery aneurysms: clinical features and outcome of 80 patients. World Neurosurg. (2014) 82:702–13. doi: 10.1016/j.wneu.2014.06.012, PMID: 24937594

[113] Shi L Xu K Sun X Yu J. Therapeutic progress in treating vertebral dissecting aneurysms involving the posterior inferior cerebellar artery. Int J Med Sci. (2016) 13:540–55. doi: 10.7150/ijms.15233, PMID: 27429591 PMC4946125

[114] Hou K Yu J. Case report: can ruptured aneurysms in the hypoplastic and plexiform posterior inferior cerebellar arteries be safely occluded? Front Neurol. (2022) 13:904863. doi: 10.3389/fneur.2022.904863, PMID: 35812084 PMC9263357

[115] Lewis SB Chang DJ Peace DA Lafrentz PJ Day AL. Distal posterior inferior cerebellar artery aneurysms: clinical features and management. J Neurosurg. (2002) 97:756–66. doi: 10.3171/jns.2002.97.4.0756, PMID: 12405360

[116] Malcolm JG Grossberg JA Laxpati NG Alawieh A Tong FC Cawley CM. Endovascular sacrifice of the proximal posterior inferior cerebellar artery for treatment of ruptured intracranial aneurysms. J Neurointerv Surg. (2020) 12:777–82. doi: 10.1136/neurintsurg-2020-016261, PMID: 32546632

[117] Hou K Guo Y Xu B Xu K Yu J. Delayed establishment of collateral circulation from posterior meningeal artery after proximal occlusion of posterior inferior cerebellar artery: case report and literature review. World Neurosurg. (2018) 115:334–7. doi: 10.1016/j.wneu.2018.04.207, PMID: 29751186

[118] Hou K Lv X Guo Y Yu J. Endovascular treatment of posterior inferior cerebellar artery trunk aneurysm. Acta Neurol Belg. (2022) 122:1405–17. doi: 10.1007/s13760-021-01826-8, PMID: 34677822

[119] Isokangas JM Siniluoto T Tikkakoski T Kumpulainen T. Endovascular treatment of peripheral aneurysms of the posterior inferior cerebellar artery. AJNR Am J Neuroradiol. (2008) 29:1783–8. doi: 10.3174/ajnr.A1218, PMID: 18635613 PMC8118767

[120] Lazaro T Vasandani V Robledo A Gadgil N Kan P. Flow diversion of a dissecting PICA aneurysm. Neurosurg Focus Video. (2022) 7:V9. doi: 10.3171/2022.7.FOCVID2247, PMID: 36425264 PMC9664496

[121] Oğuz Ş Dinc H. Treatment of posterior inferior cerebellar artery aneurysms using flow-diverter stents: a single-center experience. Interv Neuroradiol. (2019) 25:407–13. doi: 10.1177/1591019918824003, PMID: 30803331 PMC6607619

[122] Tone O Sato Y Kubota Y Takada Y. Unruptured aneurysmal shrinkage of the distal posterior inferior cerebellar artery following stent jailing of the arterial orifice: a case report. NMC Case Rep J. (2021) 8:651–6. doi: 10.2176/nmccrj.cr.2021-0090, PMID: 35079530 PMC8769393

